# CLEC16A interacts with retromer and TRIM27, and its loss impairs endosomal trafficking and neurodevelopment

**DOI:** 10.1007/s00439-022-02511-3

**Published:** 2022-12-20

**Authors:** Daphne J. Smits, Jordy Dekker, Rachel Schot, Brahim Tabarki, Amal Alhashem, Jeroen A. A. Demmers, Dick H. W. Dekkers, Antonio Romito, Peter J. van der Spek, Tjakko J. van Ham, Aida M. Bertoli-Avella, Grazia M. S. Mancini

**Affiliations:** 1 Department of Clinical Genetics, ErasmusMC University Medical Center, 3015 CN Rotterdam, the Netherlands; 2 Division of Pediatric Genetics, Department of Pediatrics, Prince Sultan Military Medical City, Riyadh, 12233 Saudi Arabia; 3 Department of Molecular Genetics, Proteomics Center, ErasmusMC University Medical Center, 3015 CN Rotterdam, the Netherlands; 4 CENTOGENE GmbH, 18055 Rostock, Germany; 5 Department of Pathology, Clinical Bioinformatics, ErasmusMC University Medical Center, 3015 CN Rotterdam, the Netherlands

## Abstract

CLEC16A is a membrane-associated C-type lectin protein that functions as a E3-ubiquitin ligase. CLEC16A regulates autophagy and mitophagy, and reportedly localizes to late endosomes. GWAS studies have associated *CLEC16A* SNPs to various auto-immune and neurological disorders, including multiple sclerosis and Parkinson disease. Studies in mouse models imply a role for CLEC16A in neurodegeneration. We identified bi-allelic *CLEC16A* truncating variants in siblings from unrelated families presenting with a severe neurodevelopmental disorder including microcephaly, brain atrophy, corpus callosum dysgenesis, and growth retardation. To understand the function of CLEC16A in neurodevelopment we used in vitro models and zebrafish embryos. We observed CLEC16A localization to early endosomes in HEK293T cells. Mass spectrometry of human CLEC16A showed interaction with endosomal retromer complex subunits and the endosomal ubiquitin ligase TRIM27. Expression of the human variant leading to C-terminal truncated CLEC16A, abolishes both its endosomal localization and interaction with TRIM27, suggesting a loss-of-function effect. CLEC16A knockdown increased TRIM27 adhesion to early endosomes and abnormal accumulation of endosomal F-actin, a sign of disrupted vesicle sorting. Mutagenesis of *clec16a* by CRISPR–Cas9 in zebrafish embryos resulted in accumulated acidic/phagolysosome compartments, in neurons and microglia, and dysregulated mitophagy. The autophagocytic phenotype was rescued by wild-type human CLEC16A but not the C-terminal truncated CLEC16A. Our results demonstrate that CLEC16A closely interacts with retromer components and regulates endosomal fate by fine-tuning levels of TRIM27 and polymerized F-actin on the endosome surface. Dysregulation of CLEC16A-mediated endosomal sorting is associated with neurodegeneration, but it also causes accumulation of autophagosomes and unhealthy mitochondria during brain development.

**Supplementary Information:**

The online version contains supplementary material available at 10.1007/s00439-022-02511-3.

## Introduction

Development of the human cerebral cortex is a complex process that is strictly regulated from embryonic development until adult age. Abnormalities during this process can result in various anomalies, including microcephaly, which is defined as a head circumference and brain size lower than −2 SD corrected for age and sex (Barkovich et al. 2012). Microcephaly can result from a shortage in neuronal progenitor numbers during neurogenesis. This deficit can be caused by insufficient proliferation or increased cell death of neuronal progenitors. Genetic factors play a major role in the pathogenesis of microcephaly and are known to interfere with the number of neuronal progenitor cells via a variety of biological processes (Jayaraman et al. 2018; Siskos et al. 2021). Most microcephaly-related genes affect the cell cycle by interfering with mitotic spindle formation, centrosome maturation, or DNA repair. However, over the last years, there has been a remarkable discovery of genes related to endoplasmic reticulum (ER) stress, autophagy, endosomal trafficking, and protein homeostasis (García-Cazorla et al. 2022; Jayaraman et al. 2018; Passemard et al. 2019).

C-type lectin protein 16A (CLEC16A) is a member of the C-type lectin (CLEC) protein family. CLEC proteins are essential in the regulation of the adaptive immune responses and recognize antigens via their carbohydrate recognition domain to guide them through the endosomal system to the surface of antigen-presenting cells. Unlike other CLEC proteins, CLEC16A lacks an active/full length carbohydrate recognition domain and functions as an E3-ubiquitin ligase involved in the regulation of autophagy and mitophagy (Kim et al. 2010, 2012; Pearson et al. 2018; Redmann et al. 2016; Soleimanpour et al. 2014). Despite the absence of a functional carbohydrate recognition domain, several GWAS studies have shown a link between CLEC16A and the human immune system by associating the *CLEC16A* locus with susceptibility to auto-immune diseases, including type-1 diabetes (Hakonarson et al. 2007; Wellcome Trust Case Control Consortium 2007), multiple sclerosis (International Multiple Sclerosis Genetics Consortium 2009; International Multiple Sclerosis Genetics Consortium et al. 2007; Nischwitz et al. 2011), primary biliary cholangitis (Hirschfield et al. 2012), Addison disease (Skinningsrud et al. 2008), juvenile idiopathic arthritis (Skinningsrud et al. 2010), and neurodegenerative disorders like Parkinson disease (Fan et al. 2022; Strafella et al. 2021). CLEC16A is thought to regulate immune tolerance via the endosome–lysosome trafficking by stimulating autophagy of thymic epithelial cells and lowering of the antigen-presenting function (Schuster et al. 2015), by the regulation of HLA class-II antigen expression via late endosome biogenesis in antigen-presenting cells and by participating in retrograde transport of HLA-II containing compartments in myeloid cells (Li et al. 2015; Rijvers et al. 2020; van Luijn et al. 2015).

The first link between CLEC16A and autophagy was made from a Drosophila study on its homologue *Ema*, which appeared to regulate endosomal maturation and autophagy through interactions with the HOPS complex (Kim and DiAntonio 2012). Later, it was shown that knockdown of CLEC16A disrupts the autophagic flux and regulates autophagy initiation via delaying mTOR (mammalian target of Rapamycin) activity (Team RC et al. 2017). CLEC16A forms a complex together with Nrdp1 and USP8, which promotes mitophagic processes and controls the mitochondrial quality (Pearson et al. 2018). Adequate regulation of mitophagy, i.e., the removal of only damaged mitochondria through autophagy, protects the cell against mitochondria-induced cell death and has been implicated in the pathogenesis of neurodegenerative disorders (Soleimanpour et al. 2015).

The function of CLEC16A in CNS is studied in mice, where it is shown to have a protective role against neurodegeneration. *Clec16a* knockout (KO) mice present with severe neurodegeneration, loss of cerebellar Purkinje cells associated with accumulated autophagosomes and activation of autophagy marker LC3 (Redmann et al. 2016). Moreover, the impaired secretory capacity of Clec16a deficient neuronal cells induces ER stress in a conditional KO *Clec16a* mouse model (Hain et al. 2021). The physiological ER stress response is essential during normal brain development by acting as a protective and intrinsic regulator of protein synthesis and metabolic homeostasis. However, increased ER stress levels lead to neurodegeneration as well as neuronal loss during embryonic development (Hetz and Saxena 2017; Murao and Nishitoh 2017; Passemard et al. 2019). In humans, the only link between *CLEC16A* and neurological disease is made by the association between its locus and susceptibility to neurological disorders, like multiple sclerosis (MS) (International Multiple Sclerosis Genetics Consortium et al. 2007; Mero et al. 2011) and Parkinson disease (Fan et al. 2022; Strafella et al. 2021). Despite these observations, the role of CLEC16A in human brain development and neurodegeneration remains poorly understood.

Here, we report two unrelated families with four affected individuals who have bi-allelic loss-of- function (LoF) variants in *CLEC16A* and recessive mode of inheritance presenting with progressive microcephaly, brain atrophy, corpus callosum hypoplasia, growth retardation, hypotonia, and severe developmental delay. We provide clinical and neuroimaging data to expand the current knowledge on the contribution of CLEC16A to human neurological disease and investigate the role of CLEC16A during brain development using both in vitro and in vivo model systems.

## Materials and methods

### Consent

The study was approved by the local IRBs (Erasmus MC Rotterdam, protocol METC-2012387). Written informed consent for participation in the study and scientific publication of imaging data and clinical photographs was obtained.

### Exome sequencing (ES)

Exome sequencing was performed on DNA isolated from blood from probands and family members, as previously described (Vandervore et al. 2019b). In individual II-3 from family 1, DNA was isolated from skin fibroblasts, as this was the only available source. Details of sequencing and analysis pipelines are described for each family.

Family 1: WES was performed on the Agilent Sure Select platform (Clinical research Exome Capture), run on HiSeq (101 bp paired-end, Illumina), using the diagnostic certified pipeline of the Department of Clinical Genetics, ErasmusMC, Rotterdam. The average coverage is ~ 50 ×. Data are demultiplexed by the Illumina Software CASAVA. Reads are mapped with the program BWA (http://bio-bwa.sourceforge.net/). Variants are detected with the Genome Analysis Toolkit (http://www.broadinstitute.org/gatk/). The Variant Calling File is filtered in Alissa Interpret.

Amplification reactions were conducted according to standard methods and purified with ExoSAP-IT (USB). Direct sequencing was performed with Big Dye Terminator chemistry (Applied Biosystems). DNA fragment analysis was performed with capillary electrophoresis on an ABI3130 Genetic Analyzer (Applied Biosystems) with the software package Seqscape (Applied Biosystems).

Family 2: DNA was extracted using standard methods from dried blood spots submitted on filter cards (CentoCard^®^). Exome sequencing was performed in a diagnostic laboratory, as described previously (Trujillano et al. 2017). Briefly, RNA capture baits against approximately 60 Mb of the human exome (targeting > 99% of regions in CCDS, RefSeq and Gencode databases) was used to enrich regions of interest from fragmented genomic DNA with the Agilent’s SureSelect Human All Exon V6 kit. The generated library was sequenced on an Illumina platform to obtain an average coverage depth of ~ 100 × . Typically, ~ 97% of the targeted bases are covered > 10 × . An end-to-end in-house bioinformatics pipeline including base calling, alignment of reads to GRCh37/hg19 genome assembly, was applied. Exon 1 (NM_001243403.1) containing the variant was amplified (primers available upon request) and Sanger sequenced in both forward and reverse direction on a 3730xl sequencer (Thermo Fisher Scientific, Waltham, MA). Variant nomenclature followed standard Human Genome Variation Society (HGVS) recommendations (den Dunnen et al. 2016).

### Data availability statement

Exome sequencing data are deposited internally in each medical institute referring the patients, in respect to the privacy of the families.

### Cell culture and plasmid transfections

All cell types used were grown in DMEM supplemented with 10% fetal bovine serum, 1% L-glutamine, and 1% penicillin/streptomycin at 37 °C and 5% CO_2_. For the expression of exogenous *CLEC16A*, human embryonic kidney 293 T (HEK293T) cells were transfected with lipofectamine 2000 (3 μl/ug plasmid) in serum-free media. 48 h after transfection, cells were fixed in 4% paraformaldehyde (PFA) for immunocytochemistry or lysed in 1% TNE buffer for immunoprecipitation.

### siRNA transfections

HEK293T cells were transfected at 60–70% confluency with *CLEC16A* siRNA siGenome smartpool Dharmacon (M-022485–02-0005) or equal amounts of siGENOME non-targetting siRNA pool #3 (siCTRL). Transfection mixes were prepared in serum-free media, containing 1.25 μl siRNA (20 µM) and 2.5 μl DharmaFECT3 transfection reagent per mL of final culture media. Cells were lysed for RNA expression 24 h after transfection. For protein analysis, cells were harvested at least 48 h after transfection.

### RT-qPCR

Fibroblasts from skin biopsies, previously sampled and stored for diagnostic purposes, were grown till 80% confluence in DMEM as described above, followed by RNA isolation using the RNeasy Mini Kit (QIAGEN). RNA was reverse transcribed with the iScript cDNA Synthesis Kit (Bio-Rad Laboratories). RT-qPCR was performed using iTaq Universal SYBR Green Supermix (BioRad) with CLEC16A-RT primers (Supplementary Table 1).

### Cloning

The pEGFP-CLEC16A-C1 and 2HA-CLEC16A plasmids (NM_001243403, kind gift from Dr. Marvin M. van Luijn, department of immunology ErasmusMC (van Luijn et al. 2015)) were transformed into semi-competent Escherichia coli XL-10 gold bacteria strains and isolated as described previously (Magini et al. 2019; Vandervore et al. 2019b). For site-directed mutagenesis (SDM) of the human *CLEC16A* sequence, the Q5 Site-Directed Mutagenesis Kit (NEB) was used following the manufacturer’s protocol. The primers used for SDM are listed in Supplementary Table 1. The variant identified in family 1 was subcloned into the pEGFP-CLEC16A-C1 plasmid. Efficiency of the procedure, which results in N-terminally tagged GFP (pEGFP-CLEC16A), was confirmed with Sanger sequencing of the entire *CLEC16A* gene construct. PCR amplification and Sanger sequencing were conducted according to standard methods.

### Immunoprecipitation and proteomics

Immunoprecipitation of exogenous pEGFP-CLEC16A was performed 48 h after transfection of a 10 cm dish with HEK293T cells. Lysis was performed with 1% TNE (1%Triton, 50 mM NaF, 100 mM NaCl, 50 mM Tris–HCl pH 7.6). Cell lysates were incubated on ice for 20 min and centrifuged at 10,000 × g for 10 min at 4 °C. Primary antibodies were added to the supernatant and incubated overnight at 4 °C while rotating. The following day, 50ul protein A agarose beads (GE Healthcare) were added to the mixture and incubated for 4 h. Mixtures were washed three times with TNE-1% lysis buffer and centrifugation at 1,000 × g for 15 s at 4 °C. For western blot analysis, beads were diluted in 4 × Laemmli buffer. The interacting proteome was determined by LC–MS/MS at the proteomics core facility of the ErasmusMC Rotterdam as described previously (Magini et al. 2019; Vandervore et al. 2019a, 2019b). Mass spectrometry data were analyzed with MaxQuant software.

### Western blot

Fifteen 5 days post-fertilization (dpf) zebrafish larvae were lysed in 35 uL 4 × Laemmli buffer containing 0.4 mM DTT for 10 min at 96 °C with regular vortexing. Cell lysates were made in RIPA buffer and diluted in 4 × Laemmli buffer containing 5% β-mercaptoethanol. Equal amounts of protein from cells or zebrafish lysates were loaded on a 4–15% Criterion TGX Stain-Free Protein Gel and separated at 150 volt for 60 min in Tris–glycine-SDS running buffer. Proteins were transferred to a nitrocellulose membrane (Amersham Protran 0.45 NC, GE Healthcare Life Sciences) for 10 min at 2.5A and 25 V. Membranes were blocked for 1 h in 0.1% PBS–Tween containing 5% low-fat milk powder (Elk, Campina). Incubation with the first antibody was performed overnight in blocking buffer at 4 °C. The following day, membranes were incubated with the secondary antibody for 1 h at room temperature (RT). Proteins were revealed with a fluorescent-based approach on the Odyssey Infrared Imager (LI-COR Biosciences).

### Immunofluorescence

HEK293T cells were fixed with 4% PFA for 20 min on ice. Samples were blocked on ice for 1 h in blocking buffer containing 50 mM Tris HCl [pH 7.4], 0.9% NaCl, 0.25% gelatin, and 0.5% Triton X-100. Primary antibodies were dissolved in blocking buffer. Sections were incubated overnight at 4 °C. The next day slides were washed three times with PBS and incubated with the secondary antibodies for 1 h at RT. Coverslips were mounted with DAPI containing Prolong Gold.

### Antibodies

Primary antibodies: mouse anti-EEA1 ( ICC 1:300, novus biologicals 6D4.1D4), mouse anti-LAMP2 (1:200 BD biosciences 555803), rabbit anti-GFP (IP 1:100 abcam AB290), mouse anti-VPS35 (ICC 1:200, IP 1:50 Santa Cruz, sc-374372), rabbit anti-RET finger protein/TRIM27 (ICC 1:200, IP 1:50, IBL18791), rabbit anti-Parkin (WB 1:1000, novus biologicals NBP2-41287), rabbit anti-HA-Tag (ICC 1:800, cell signaling technologies, C29F4).

Secondary antibodies used for ICC: green goat anti-rabbit IgG (H + L)488 (1:200, Thermo Fisher Scientific, A11088) and Cy3 AffiniPure donkey anti-mouse IgG (H + L) (1:200, Jackson Laboratories, 715–165-150), donkey anti-rabbit cy3 (H + L) (1:200, Jackson Laboratories 711–165-151), phalloidin 594 1:40 (Invitrogen A12381).

Secondary antibodies used for WB (1:10 000): red IRDye 680RD goat anti-rabbit IgG (H + L) (LI-COR Biosciences, 926–68,071) and green IRDye 800CW goat anti-mouse IgG (H + L) (LI-COR Biosciences, 926–32210).

### Fish care

In this study, the following zebrafish line were used: Tg(mpeg1:GFP) and Tg(slc1a2b:Citrine) (Ellett et al. 2011; He et al. 2009; Kuil et al. 2019a). Zebrafish embryos were kept on a 14 h/10 h light–dark cycle at 28 °C in E3 medium buffered with 20 mM HEPES pH 7.2. E3 medium was refreshed at 24 h post-fertilization (hpf) with E3 containing 0.003% 1-phenyl 2-thiourea (PTU) to prevent pigmentation.

### LysoTracker staining zebrafish

Fifteen zebrafish larvae at 3 and 5 dpf of both control and *clec16a* crispants were transferred to a round bottom 2 ml Eppendorf tube. E3 medium was carefully removed as much as possible and 200 µl LysoTracker™ Red DND-99 (Invitrogen, L7528) diluted 1:100 (final concentration 10uM) in E3 containing 0.003% PTU was added to the fish. Fish were incubated in the dark with tube caps open at 28 °C for 40 min. Next, larvae were washed 2 × with E3 medium containing 0.003% PTU and incubated for 20 min at 28 °C before imaging.

### CRISPR–Cas9 injections into zebrafish 1-cell stage for crispant zebrafish

crRNA were designed with the The Alt-R™ CRISPR–Cas9 System of IDT. *clec16a* crRNA targeting exon 4 sequence: 5′-TGATTTTGCCCTGTACACGG-3′ and control crRNA sequence: 5′- 5′-AACGATACCATGGTGAGCAA-3′. *clec16a* crRNA was designed equal to the coding strand to prevent possible annealing to *clec16a* mRNA in the rescue experiment. Equal volumes of tracrRNA in duplex buffer (IDT) and crRNA were incubated at 95 °C for 5 min, subsequently cooled down at RT resulting in a 50 µM cr:tracrRNA complex. SpCas9 was synthesized using the SP-Cas9 plasmid (Addgene plasmid #62,731) as described previously (D'Astolfo et al. 2015). 50 pmol cr:tracrRNA was mixed with 25 pmol SpCas9 in a total volume of 3 µl and incubated at RT for 5 min to form cr:tracrRNA-Cas9 RNPs. Subsequently, 3 µl 300 mM KCl and 0.3 µl was added to the mix and 1 nl was injected in the cell of a 1-cell stage embryo to generate crispant zebrafish with a high indel frequency.

### Genotyping larvae

After imaging, zebrafish larvae were euthanized and placed in single tubes containing 40 µl 50 mM NaOH and incubated at 95 °C for 30 min for lysis. Next, samples were cooled down briefly on ice and 4 µl 1 M Tris–HCl pH8 was added. 1 µl of lysis was used for PCR using FastStart™ Taq polymerase (Sigma-Aldrich) under following conditions: 95 °C for 5 min, followed by 35 cycles of 94 °C for 30 s, 58 °C for 30 s, 72 °C for 45 s, finished by a final step of 72 °C for 5 min. 5 µl PCR product was visualized on a Tris–borate–EDTA (TBE) 2% agarose gel. Next, PCR samples were selected for Sanger sequencing. Indel efficiency and frequency were calculated using the online tool TIDE (Brinkman et al. 2014).

### Neutral red staining

Microglia were labeled by incubation of 3 and 5 dpf zebrafish larvae in E3 medium containing 2.5 µg/ml neutral red (Sigma-Aldrich) and 0.003% PTU for 2 h at 28 °C. After staining, zebrafish larvae were washed with E3 medium containing 0.003% PTU and incubated at 28 °C for 30 min prior imaging.

### MitoTracker staining of zebrafish and analysis

Twelve zebrafish larvae of both control and *clec16a* crispants were transferred to a twelve-wells dish. E3 medium was removed and 2 ml MitoTracker™ Red CMXRos (Invitrogen, M7512) diluted 1:1000 in E3 medium containing 0.003% PTU was added to the fish at 4 dpf. Incubation with MitoTracker was done overnight in the dark for 16 h, followed by 2 × wash with E3 containing 0.003% PTU and another incubation in only E3 containing 0.003% PTU for 20 min at 28 °C in the dark before imaging at 5 dpf. Analysis of the MitoTracker signal was done by taking a region of interest containing the radial fibers of the right hemisphere in the midbrain region. The same threshold for each independent experiment was applied to each image, to assure only the regions with the highest intensity of the MitoTracker signal were counted. The area of the high intensity MitoTracker staining was divided by the total area of the image to correct for image size.

### Rescue experiment in zebrafish with human CLEC16A mRNA

Forward primer were designed over Kozak sequence and ATG start codon containing a T7 promoter tail. Reverse primer was designed containing the stop codon (Supplementary Table 1). Subsequently a linear dsDNA template was generated by PCR using the human WT CLEC16A plasmid or the CLEC16A-Δ19 plasmid as template. dsDNA templates were checked on a 1% TBE agarose gel. Purification of the dsDNA template was performed by cutting the correct band out of gel and isolate the DNA product using the nucleospin gel and PCR Clean up Kit (Macherey–Nagel). From the purified dsDNA template, an in vitro mRNA synthesis was performed using the mMESSAGE mMACHINE™ T7 Transcription Kit (Invitrogen) according to manufacturer’s protocol. mRNA isolation and DNA removal was performed according to the mMESSAGE mMACHINE™ T7 Transcription Kit manufacturer’s protocol; purified mRNA were dissolved in nuclease-free H_2_O. Zebrafish 1-cell stage embryos were first injected with 1 nl cr:tracrRNA-Cas9 RNPs as described above, followed by 3 nl (= 3 pg) human *CLEC16A*-WT mRNA and human *CLEC16A*-Δ19 mRNA and nuclease-free H_2_O as control.

### Imaging

Confocal fluorescent images of cells were acquired with the Broadband Leica TCS SP5, using Leica LAS AF software (Leica Microsystems). Lasers with 405 nm, 488 nm, and 561 nm excitation wavelength were used to visualize DAPI, GFP, and cy3/alexa488 secondary antibodies.

Super-resolution 3D-SIM microscopy was used to assess localization of pEGFP-CLEC16A to endosomes. Images were made with Elyra Zeiss PS1. 3D-SIM, using a 63 × 1.4NA oil objective. The grating present in the light path was modulated in five phases and five rotations. Multiple z-slices were recorded on an Andor iXon DU 885, 1002 × 1004 EMCCD camera. Reconstruction of the images was performed with the Zeiss Zen software.

For imaging of zebrafish larvae, the larvae were anesthetized with 0.016% MS-222 and imbedded in 1.8% low melting point agarose (Invitrogen). Three to five images in the z plane were acquired with the Leica M165 FC microscope using the 12 × dry objective and a Leica DFC550 camera. For confocal imaging, zebrafish larvae were imbedded as described above. Confocal imaging was performed at a Leica SP5 intravital microscope with a 20 × /1.0 NA water-dipping lens. Imaging of GFP was performed with the 488 nm laser, Citrine the 514 nm laser, and LysoTracker/MitoTracker the 561 nm laser.

All images were processed and blindly analyzed with Fiji ImageJ software. For the quantification of endosomal F-actin intensity, images were thresholded on the VPS35 signal. The mean fluorescent intensity of F-actin was measured in the thresholded region only and corrected for F-actin background levels.

### Interactome analysis

All interactors with an Andromeda score > 40 were analyzed using the online Database for Annotation, Visualization, and Integrated Discovery (DAVID) Bioinformatics Resources v.6.7 and the STRING V11.5 platform (Szklarczyk et al. 2019). Replicates with only one identified peptide were excluded from the analysis. Protein clusters were made with *K*-means clustering. The *K* value for this analysis was set at 4.

### Statistics

All statistical tests were performed with Prism GraphPad 9 Software. All data sets were tested for outliers and normality. *p* values < 0.05 were considered significant. All error bars represent the standard error of the mean (SEM). Details about the statistical tests are available in figure legends.

## Results

### Clinical phenotype

We identified infants with *CLEC16A* variants in two families. Extensive queries in international databases Genematcher, Genome England, Centogene Gmbh Rostock, and institutes databases of members of the Neuro-MIG consortium (COST Action CA16118, www.cost.eu/actions/CA16118) failed to identify additional cases with the same inheritance pattern. Exome sequencing (ES) was performed in probands from these two unrelated consanguineous families with a severe neurodevelopmental disorder. A summary of all clinical features observed in the affected individuals is available in Supplementary Table 2.

**Fig. 1:**
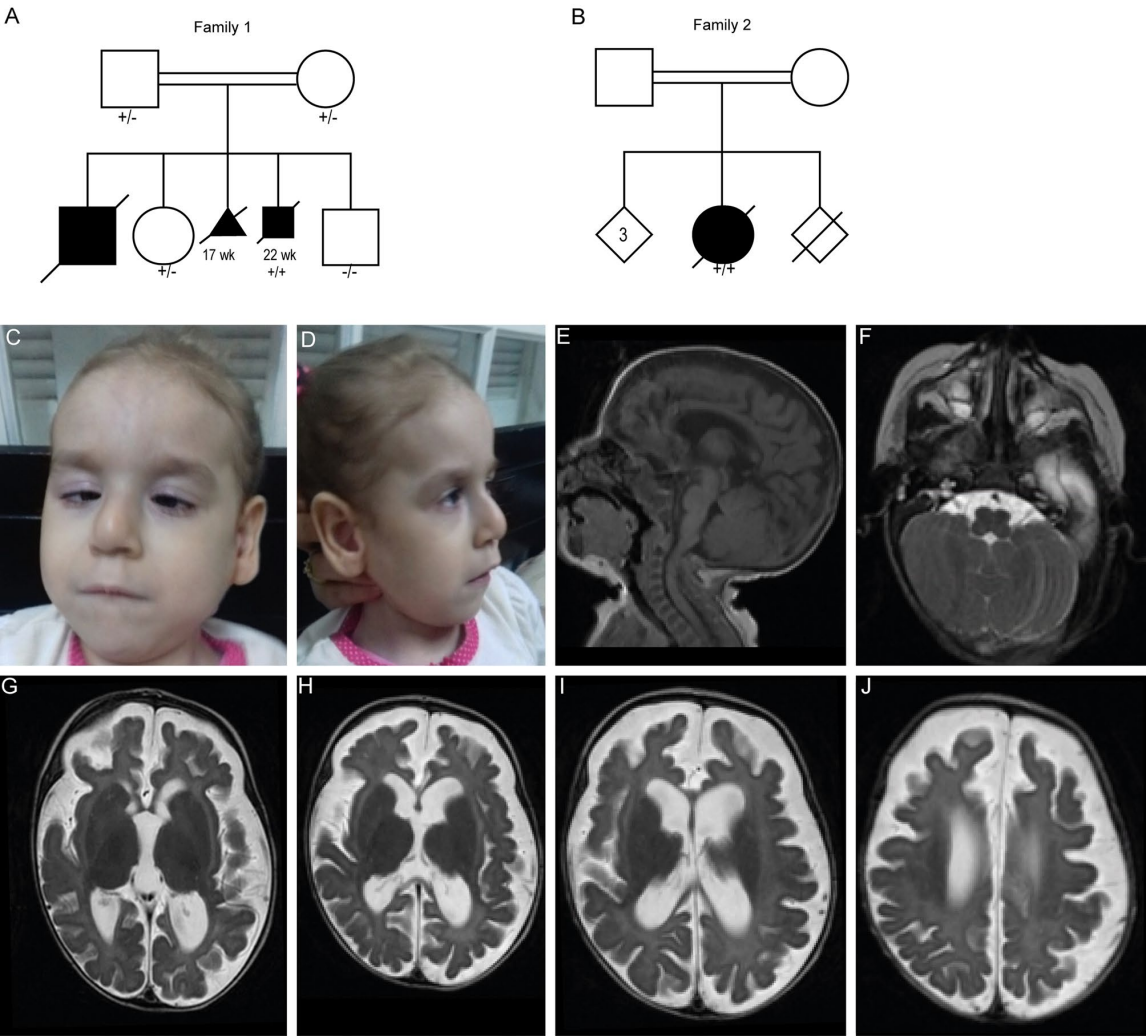
Clinical features and brain MRI of individuals with *CLEC16A* variants. **a**,** b** Pedigrees of the two families with *CLEC16A* variants. Black boxes represent affected individuals. + / − indicate heterozygous individual, + / + indicates homozygous individual, − / − indicates homozygous WT. Subjects II*-*1/2 from family 1 presented with similar clinical features as observed in individual II-3 but were not tested for the *CLEC16A* variant, as there was no DNA available for these siblings. Heterozygosity was confirmed for both parents. **c**, **d** Facial features of subject II-1 from family 2. **e**–**j** Brain MRI of the affected individual of family 2 made at 3 months of age with midsagittal T1-weighted images (**e**), and axial T1-weighted images

The first family consists of three affected and two healthy siblings born from healthy consanguineous parents (Fig. 1a). The first sibling (fam 1 II-1) was born in 1987. The pregnancy was uncomplicated until the sixth month after which growth delay of the fetus was noted. The boy was born at term, microcephalic and was small for the gestational age [weight 2.3 kg (− 2.5SD), length 45 cm (− 2SD), head circumference (HC) 29.5 cm (− 3.5SD)]. After birth, he presented with progressive microcephaly and growth retardation. At 4 months his weight was 5 kg (− 2SD), length 50 cm (− 5.5SD) and HC 34 cm (− 5.5SD), he had feeding difficulties, failure to thrive, constipation, vesicoureteral reflux grade II, and cryptorchidism. Physical examination at 4 months of age showed a low set hairline, large ears, brachymesophalangia of the fifth finger, bilateral vertical talus, hypotonia, tetraplegia, inappropriate eye contact, and absence of responds to sound. Cranial ultrasound at birth and postnatal cranial CT reported dilatation of both central and peripheral liquor spaces and hypoplasia/agenesis of the corpus callosum, no calcifications. Brain auditory evoked responses showed neurosensory hearing loss. In the first weeks after birth, he started to have recurrent infections, including pneumonia and recurrent sepsis, from which he passed away at 5 months of age. From this sibling there is no material available for further (genetic) analysis.

The third (fam 1 II-2) and fourth (fam 1 II-3) pregnancies were also monitored by US and terminated at 17 weeks and 22 weeks of gestation, respectively, due to microcephaly, IUGR, hydrocephalus, and suspected agenesis/hypoplasia of the corpus callosum. Autopsy on the lastborn fetus (II-3) confirmed agenesis of the corpus callosum. Fibroblasts derived from this fetus were tested for pyruvate dehydrogenase (PDH) activity, DNA repair capacity, cholesterol metabolism, and peroxisomal very long-chain fatty acid oxidation. No abnormalities were found during these investigations. The second and the fifth pregnancies were uneventful and ended with the birth of healthy children.

The second family consists of three healthy siblings, one deceased sibling (unexplained death at 4 months of age), and one affected female, all born from consanguineous parents of Arab ancestry (Fig. 1b). The affected sibling was born at 34 weeks of gestation with growth measurements conform the gestational age. From birth she presented with severe central hypotonia, peripheral hypertonia with pyramidal irritation (brisk deep tendon reflexes, positive Rossolimo sign), failure to thrive, severe developmental delay, microphthalmia and dysmorphic facial features including smooth philtrum, hypotelorism, large ears, squint, bulbous nose, thin lips, long face (Fig. 1c, d). Additionally, she suffered from recurrent urinary tract infections and mild vesical-urethral reflux. Generalized tonic clonic seizures epilepsy was first noticed at the age of 4 months and was controlled with phenobarbital. EEG recordings showed slow background activity with few discharges in the central area. Visual evoked potential and electroretinography testing did not show abnormalities. During last examination at 2 years of age, she did not reach head control, did not speak, nor walk, make eye contact or other social interactions. At that time, a severe growth delay was reported [weight 7.6 kg (− 3SD), height 72 cm (− 4SD), head circumference 41 cm (− 4SD)]. Brain MRI made at 3 months of age showed global brain atrophy, hypoplasia of the corpus callosum, and microcephaly (Fig. 1e–j). At 2 years of age, a repeat MRI showed similar findings. She died at 6 years of age from aspiration pneumonia and sepsis.

### Genomic analysis

Family 1: DNA isolated from family 1 II-3 fetal skin fibroblasts showed a normal male hybridization pattern at microarrays and many large regions of homozygosity (ROH), of which a few were not overlapping with the unaffected siblings. Diagnostic trio exome sequencing did not result in the identification of pathogenic variants. Analysis of this data in research setting, using broader splice site thresholds, identified a homozygous variant affecting a splice donor site in *CLEC16A* (NM_001243403.1 (*CLEC16A*):c.2062 + 5G > A). All other variants identified during this analysis are listed in Supplementary Table 3. *CLEC16A* is located within one of the larger regions of homozygosity. Segregation analysis confirmed heterozygosity for the variant in both parents and the healthy sister. The healthy brother does not have the variant.

Splice prediction programs (Splice-site finder, MaxEntScan, NNSPLICE, Genesplicer) predict that the variant abolishes the splice donor site of exon 19. RT-PCR around *CLEC16A* exon 19 on RNA isolated from fibroblasts, showed a shorter CLEC16A transcript (Supplementary Fig. 1a). Sanger sequencing confirmed that the shortening of this transcript is caused by the deletion of exon 19, which results in a frameshift and a premature stop codon at position p.768 (p.(Asn688Argfs*80), Supplementary Fig. 1b–d). The gnomAD (v.2.1.1) frequency of this variant is 0.00042%, but it was not reported in homozygous state.

Family 2: DNA isolated from blood showed an unremarkable karyotype and chromosomal microarray. Whole exome sequencing analysis showed a homozygous 16 base pair deletion in *CLEC16A* (NM_001243403.1 (CLEC16A): c.-4_12delCGACATGTTTGGCCGC, p.(Met1?)), which was confirmed by Sanger sequencing and is not reported in gnomAD. No other relevant variants were detected. Interestingly, the *CLEC16A* deletion eliminates the ATG initiation site and part of the corresponding Kozak consensus sequence. The human *CLEC16A* encodes two main transcript isoforms: a longer transcript containing all 24 exons (NM_015226.3) and a shorter transcript of 21 exons (NM_001243403.1). The deletion in this individual prevents translation from both isoforms. Tools predicting the use of alternative translation start sites (ATGpr) in the main transcripts showed that all other potential translation start sites have much lower probability scores, and will result in a protein lacking a significant part of the N-terminus (Supplementary Table 4). The use of alternative start sites could not be excluded completely since there is no material available from this individual for further genetic analysis. No other clinically relevant variants were identified in this individual.

### CLEC16A localizes to early endosomes

The *CLEC16A* gene is expressed in all brain regions of the adult human brain (Supplementary Fig. 2a). In single-cell RNA sequencing data derived from various human cell types, *CLEC16A* showed high expression in inhibitory and excitatory neurons, but also in astrocytes, oligodendrocytes, microglia, and spermatids (protein atlas, Supplementary Fig. 2b). Data from the human Allen brain atlas (https://portal.brain-map.org/) shows expression of *CLEC16A* during all stages of human brain development, both during embryonic stages and adulthood (Supplementary Fig. 2c). This data is compatible with a role of *CLEC16A* in brain development.

**Fig. 2:**
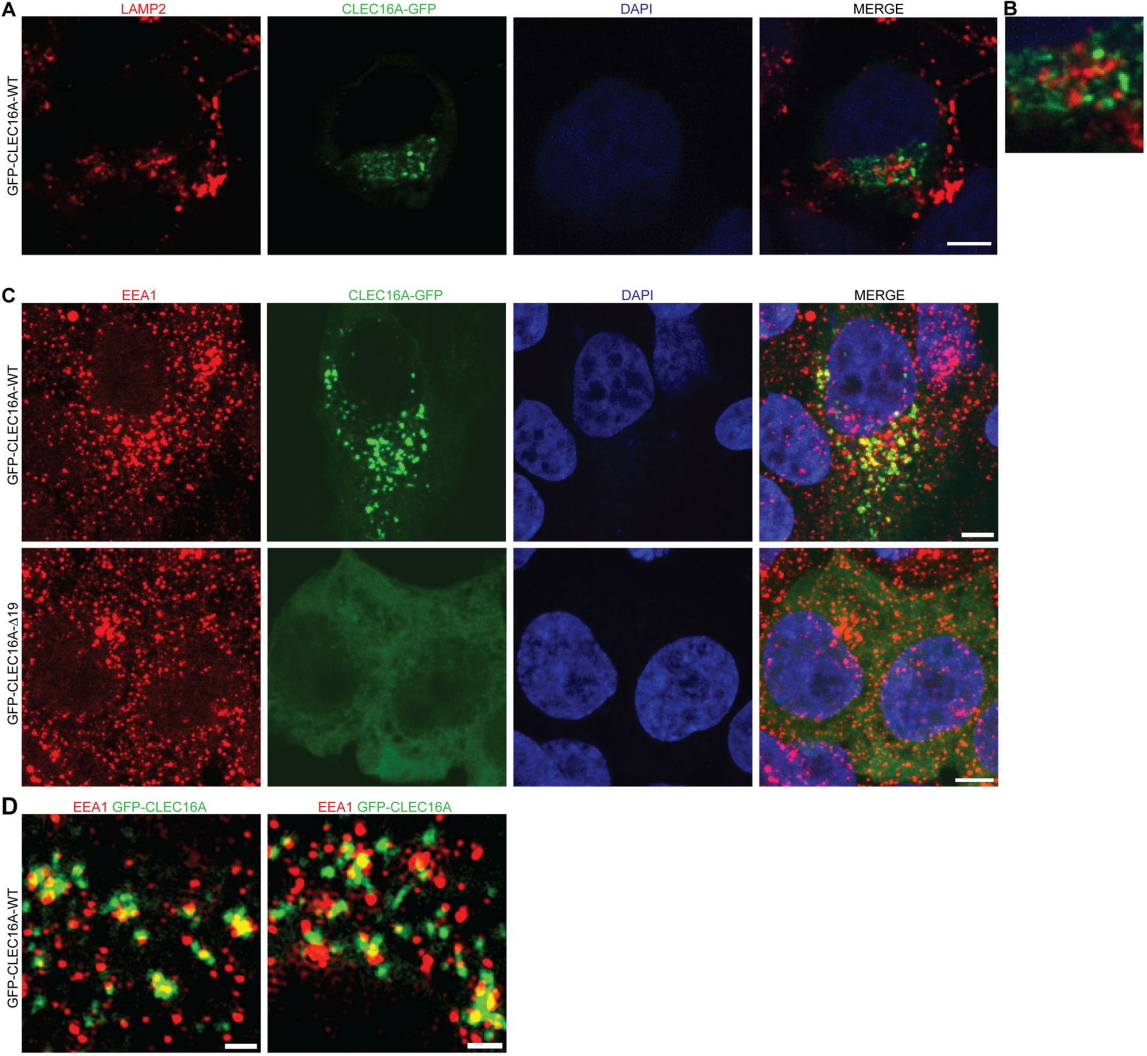
Subcellular localization of CLEC16A-WT and -Δ19. **a** Immunocytochemistry assessing co-localization of transiently expressed pEGFP-CLEC16A-WT (green) with the late endosomal/lysosomal marker LAMP2 (red). Nuclei were counterstained with DAPI. **b** Partial zoom of **a**, showing 3.5 × enlargement. **c**, **d** Immunocytochemistry assessing the localization of transiently expressed pEGFP-CLEC16A-WT (green, upper panel) and pEGFP-CLEC16A-Δ19 (lower panel) in HEK293T cells. Cells were stained for the early endosomal marker EEA1 (red). Nuclei were counterstained with DAPI. Overview images were made with confocal microscopy (**c**) and high-resolution images were with 3D-SIM super-resolution microscopy (**d**). Two representative images of pEGFP-CLEC16A-WT made with 3D-SIM super-resolution microscopy are shown in **d**. Scale bars represent 5 µm (**a**, **c**) and 1 µm (**d**)

To gain insight into the role of CLEC16A during human development, its subcellular localization was assessed by fluorescence microscopy of HEK293T cells expressing WT pEGFP-CLEC16A. Since previous studies described a role for CLEC16A in endosome–lysosome trafficking and particularly in the maturation of late endosomes into autophagosomes, we first assessed co-localization with the late endosomal/lysosomal marker LAMP2 (Kim and DiAntonio 2012; Redmann et al. 2016; van Luijn et al. 2015). Unexpectedly, pEGFP-CLEC16A showed almost no localization in proximity of LAMP2-positive vesicles (Fig. 2a, b). Instead, co-staining with the early endosomal marker EEA1 showed co-localization with CLEC16A, suggesting that CLEC16A localizes to early endosomes under these conditions (Fig. 2c). High-resolution 3D-SIM fluorescent microscopic images confirmed that a proportion of the EEA1 + endosomes are accompanied by pEGFP-CLEC16A signal on their surface, which suggests that the main localization of CLEC16A in HEK293T cells, under standard fed conditions, is on early endosomes (Fig. 2d). Localization to early endosomes was confirmed also using a HA-tagged CLEC16A construct, which excludes an artificial localization effect of the GFP tag (Supplementary Fig. 3).

To better understand the pathogenic effects of the variant identified in family 1 (p.Asn688Argfs*80), the deletion of exon 19 was introduced into the pEGFP-CLEC16A backbone by site-directed mutagenesis. This plasmid produces a CLEC16A protein lacking the C-terminal domain (Δ19 pEGFP-CLEC16A). The C-terminal domain includes a part of the putative intrinsically disordered region (IDR), but no other annotated protein domains (Gingerich et al. 2021). Overexpression of Δ19 pEGFP-CLEC16A showed synthesis of a mutant protein with a diffuse GFP signal throughout the cytoplasm, but complete loss of the early endosomal localization (Fig. 2c lower panel). These data support the pathogenicity of this variant and show that the C-terminal part of CLEC16A is essential for its endosomal localization.

### CLEC16A interacts with TRIM27 and retromer complex components

To further understand the physiological function of CLEC16A and its association with early endosomes, the interacting proteome of WT CLEC16A was assessed in HEK293T cells. Immunoprecipitation of exogenous CLEC16A-WT, followed by LC MS/MS, identified 452 reproducible peptides as putative interactors (Supplementary Table 5). We identified TRIM27 and NRDP1 as the most significant interactors of CLEC16A. NRDP1 is a known CLEC16A interactor, and their interaction regulates the induction of mitophagy (Pearson et al. 2018). TRIM27 is known to be recruited to early endosomes by the retromer complex, but the interaction with CLEC16A has not been described previously. In addition to TRIM27, we also found retromer core components VPS35 and VPS26b as potential interactors.

Subcellular compartment analysis of all interacting peptides with an Andromeda Score > 40 (199 proteins) by the DAVID platform confirmed enrichment for retromer complex signaling, which included the protein TRIM27, VPS35, VPS26b, and RAB7a. Moreover, this analysis showed enrichment for mitochondrial proteins, the proteasome complex, and the nuclear pore complex (Supplementary Table 6). Additional functional protein clustering (K-means clustering) of all identified peptides with the STRING tool resulted in four significant protein clusters (Supplementary Fig. 4a). Cellular compartment enrichment analysis on these clusters showed significant enrichment for the retromer complex (Fig. 4a), the cytosolic proteasome complex (green), the mitochondrial succinate CoA ligase complex (red), and the MMXD complex (blue), which is in line with the results from the DAVID platform analysis (Supplementary Table 6).

**Fig. 3:**
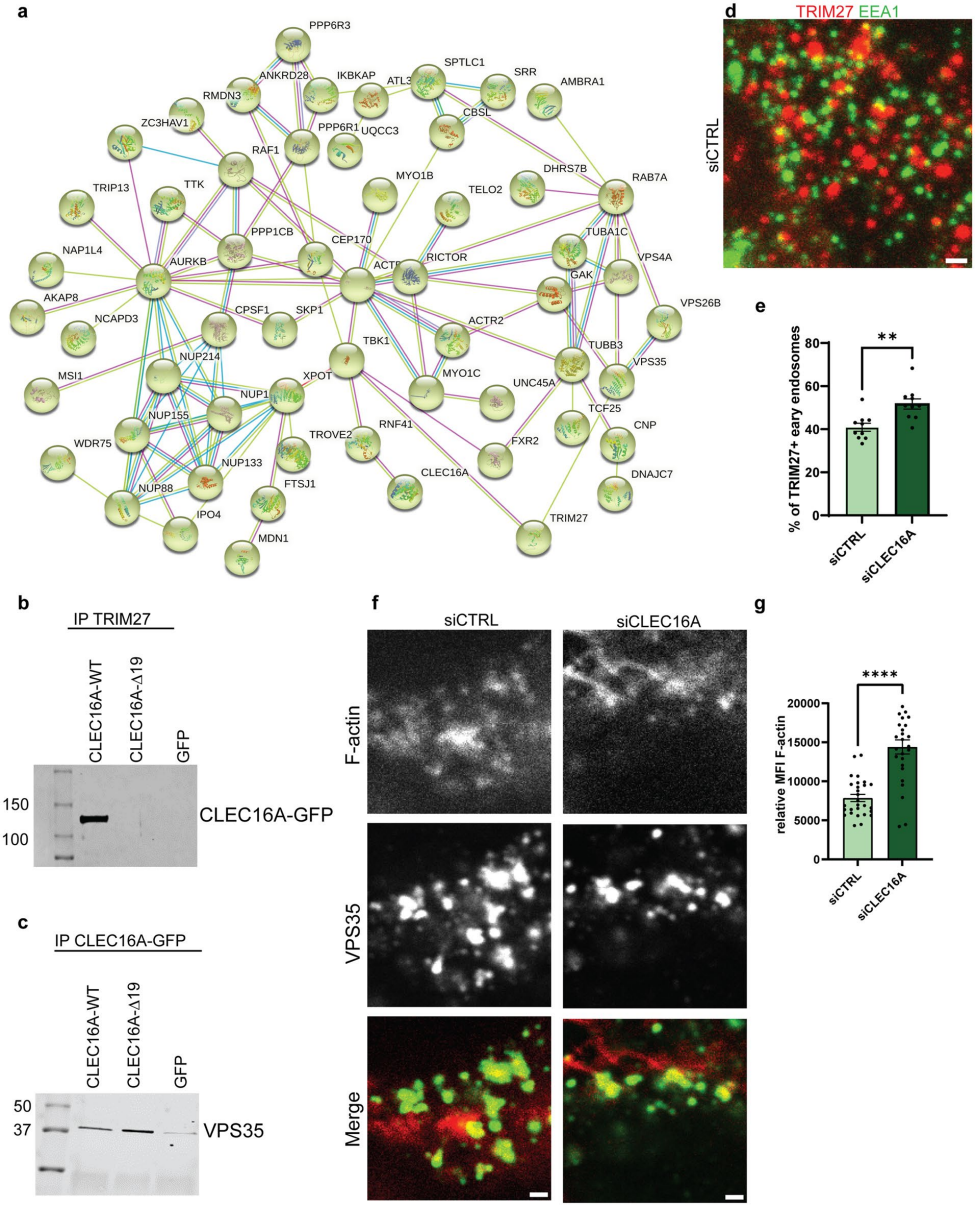
CLEC16A interactome and endosomal F-actin levels. **a** HEK293T cells were transfected with pEGFP-CLEC16A-WT, pEGFP-CLEC16A-Δ19, or negative control GFP-C1 for 48 h, followed by immunoprecipitation with anti-rabbit GFP antibodies and LC–MS/MS (*n* = 2 for all groups). Proteins interacting with pEGFP-CLEC16A-WT or pEGFP-CLEC16A-Δ19 but not with GFP-C1 were filtered based on their Andromeda score (> 40). Panel **a** shows the most significant network, which is related to the retromer complex. The colors of the lines indicate the stringency of the interaction. Light blue: from curated databases, pink: experimentally determined, green: gene neighborhood, red: gene fusions, dark blue: gene co-occurrence, yellow: text miming, black: co-expression, purple: protein homology. **b** The interaction between pEGFP-CLEC16A-WT/Δ19 was confirmed after reciprocal IP with anti-TRIM antibodies 48 h after overexpression with pEGFP-CLEC16A-WT, pEGFP-CLEC16A-Δ19, or GFP. All plasmids were revealed on immunoblot with anti-GFP antibodies. **c** The binding of pEGFP-CLEC16A-WT and Δ19 to the retromer component VPS35 was confirmed on immunoblot 48 h after transfection with pEGFP-CLEC16A-WT, pEGFP-CLEC16A-Δ19, or GFP, followed by immunoprecipitation. **d** Immunostainings on HEK293T cells treated with siCTRL or siCLEC16A siRNAs 48 h after transfection. Cells were stained with antibodies against EEA1 (green) and TRIM27 (red). **e** Quantification of **d**, unpaired *t* test with Welch’s correction (*n* = 2 experiments; *n* = 12–15 fields/section, > 150 endosomes per field counted). **f** Immunostainings on HEK293T cells treated with siCTRL or siCLEC16A siRNAs 72 h after transfection. Cells were stained with antibodies against TRIM27 (green) and phalloidin-alexa 647 (red). **g** Quantification of **f**, unpaired *t* test with Welch’s correction (*n* = 3 experiments, *n* = 10 fields/section, 3–5 regions per field analyzed). All scale bars in this Fig. represent 1 µm. **p < 0.01, ****p < 0.0001

The interactome of pEGFP-CLEC16A-Δ19 was assessed in parallel with pEGFP-CLEC16A-WT. From the 452 peptides identified in the pEGFP-CLEC16A-WT experiment, the mutant protein lost 130 peptides in both replicates. While interactions with NRDP1 and retromer components VPS35 and VPS26 were maintained, the association with the most significant interactor TRIM27 was completely lost (Supplementary Table 5). Other proteins that did not bind the truncated CLEC16A protein are mainly components of the nuclear pore complex and (nuclear) membranes (Supplementary Table 7). The specificity of the interaction between WT CLEC16A and TRIM27 was confirmed by reciprocal IP of TRIM27 and detection of pEGFP-CLEC16A-WT/Δ19 on immunoblots (Fig. 3b and Supplementary Fig. 4b). Binding of pEGFP-CLEC16A-WT and -Δ19 to retromer complex component VPS35 was confirmed in an independent experiment after immunoprecipitation of pEGFP-CLEC16A-WT/Δ19 or GFP-only and detection of VPS35 on immunoblots (Fig. 3c and Supplementary Fig. 4c). Altogether, these analyses show that TRIM27 is a strong interactor of CLEC16A in HEK293T cells, an interaction that is lost by the human C-terminal truncated ∆19 variant.

### CLEC16A depletion affects TRIM27 levels and enhances endosomal F-actin levels

We studied the relevance of the interaction between CLEC16A and TRIM27, by examining whether CLEC16A is involved in the recruitment of TRIM27 to early endosomes. We detected co-localization of early endosomal marker EEA1 with TRIM27. The number of early endosomes that showed TRIM27 signal on their surface was counted in HEK293T cells after transfection with siCLEC16A or siCTRL. *CLEC16A* expression was reduced by 75% on average after siRNA treatment (Supplementary Fig. 4d). In siCTRL HEK293T cells, 41% of the analyzed early endosomes did contain a TRIM27 positive signal on the outside of their membranes (Fig. 3d). Interestingly, this percentage increased to 52% after knockdown of CLEC16A (Fig. 3e). To confirm that these results are not due to general alterations in TRIM27 levels in *CLEC16A* depleted cells, the average fluorescent intensity of TRIM27 protein and *TRIM27* RNA levels were measured, but remained unaffected (Supplementary Fig. 4e, f). These data show that CLEC16A is not involved in recruitment of TRIM27 to early endosomes, but its presence affects the level of TRIM27 on endosomal membranes.

Early endosomes recruit TRIM27 to facilitate retromer-dependent endosomal recycling by activation of the WASH complex via various ubiquitination steps. Activation of the WASH complex finally results in nucleation of F-actin, which is needed for retromer-dependent retrograde transport (Hao et al. 2013, 2015). To assess whether the observed accumulation of TRIM27 on early endosomes in the absence of CLEC16A affects the production of F-actin, the levels of F-actin on VPS35 (retromer)-positive vesicles were measured and corrected for F-actin background levels (Fig. 3f). *CLEC16A* knockdown resulted in a significantly increased F-actin intensity on VPS35-positive vesicles (Fig. 3g, mean fluorescent intensity (MFI) siCTRL = 7856; siCLEC16A 14,396). These results suggest that CLEC16A levels affect endosomal F-actin accumulation on early endosomes by regulating endosomal TRIM27 levels.

### Clec16a depletion in zebrafish results in accumulated autolysosomes and dysregulated mitophagy

To investigate the consequences of CLEC16A loss during early brain development in vivo*,* a *clec16a* zebrafish model was generated. The zebrafish Clec16a shares 69.6% amino acid homology with the human protein. CRISPR/Cas9 and gRNA complexes were developed to mutate the zebrafish *clec16a* orthologue in zygotes (Supplementary Fig. 5a) (Kuil et al. 2019b). CRISPR/Cas9-generated mutants (crispants) were analyzed for mutagenesis efficiency, showing > 95% loss of the WT allele in ~ 94% of the embryos tested, allowing rapid LoF studies in injected zebrafish larvae (Supplementary Fig. 5b, c). *clec16a* crispants show normal general and brain morphology during the first 5 days post-fertilization (dpf). Measurements of brain width and total body length at 3 and 5 dpf did not differ from control gRNA-injected embryos (Supplementary Fig. 6a–d).

**Fig. 4:**
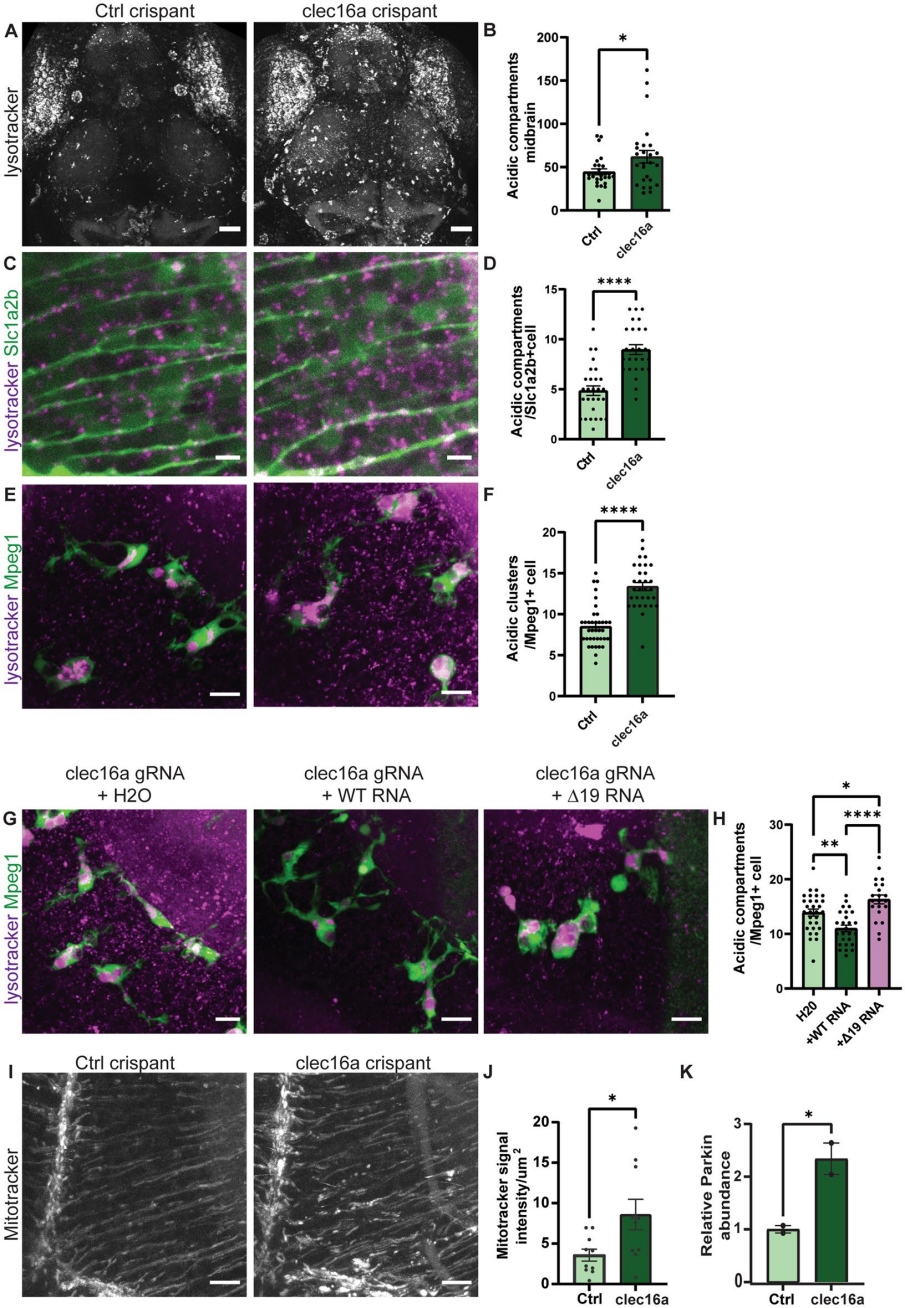
Quantification of autophagy and mitophagy in *clec16a* crispants. **a** Acidic compartments stained with LysoTracker in the *clec16a* crispants and control injected embryos at 5dpf. Scale bars indicate 50 µm. **b** Quantification of acidic compartments > 5 µm in the optic tectae of the *clec16a* crispants brain quantified. Unpaired *t* test with Welch’s correction (*n* = 2 experiments, *n* = 25 fish per group). **c** LysoTracker staining (magenta) in *slc1a2b*:Citrine background (green), visualizing radial glia. Scale bars indicate 5 µm (number of fish: *n* = 12; *clec16a* crispants *n* = 13, 2–3 cells per brain were counted, *N* = 2 experiments). **d** Quantification of **c**, unpaired *t* test with Welch’s correction. **e** LysoTracker staining (magenta) in *mpeg1*:GFP background (green), visualizing microglia (*n* = 11 fish for both groups, 3–4 cells per brain were counted, *N* = 2 experiments). Scale bars indicate 10 µm. **f** Quantification of **e**, Mann–Whitney *U* test. **g, h** Single-cell embryos were simultaneously injected with a crRNA targeting *clec16a* + *CLEC16A*-WT or *CLEC16A*-Δ19 mRNA or H_2_O (number of fish: *CLEC16A*-WT *n* = 12, *CLEC16A*-Δ19 n = 12, H_2_O *n* = 11, 2–3 cells per brain were counted, *N* = 2 experiments). Scale bars indicate 10 µm, one-way Anova. **i** MitoTracker staining of the right hemisphere midbrain region. **j** Quantification of **i**, total high MitoTracker intensity area was quantified in the radial fibers. Scale bars indicate 10 µm (*n* = 10 fish for both groups, *N* = 2 experiments, unpaired *t* test with Welch’s correction). **k** Intensities of the Parkin protein band were quantified from immunoblot (Supplementary Fig. 7a) and corrected for total protein (Supplementary Fig. 7b). The average relative Parkin abundance of the two control samples was set as a relative abundance of 1 (unpaired *t* test). *p < 0.05, **p < 0.01, ****p < 0.0001

Unlike humans, zebrafish possesses the ability to regenerate neurons throughout life (Kizil et al. 2012). Therefore, cellular changes in *clec16a* crispants were assessed despite their normal brain size. Several studies have shown the involvement of CLEC16A in endosomal trafficking and autolysosomal clearance, with accumulation of autophagosomes in Purkinje neurons of adult *Clec16a* knockout mice (Hain et al. 2021; Redmann et al. 2016). In addition, defective retromer-dependent trafficking results in accumulated autolysosomes (Seaman 2021). Therefore, we used confocal in vivo imaging of LysoTracker, a fluorescent dye which labels acidic organelles, including lysosomes and autophagosomes, in developing zebrafish brain, as performed previously in zebrafish models for membrane trafficking related disorders (Sanderson et al. 2021). In vivo confocal imaging revealed a remarkable increase of LysoTracker-positive structures in the *clec16a* crispants midbrain at 5 dpf compared to control injected fish, reminiscent of abnormal autophagocytosis (Fig. 4a, b, ctrl = 44.5, *clec16a* = 62) (Kuil et al. 2019a; Sanderson et al. 2021). Analysis at 3 dpf did not show differences between *clec16a* crispants and control larvae, which suggests a progressive effect of Clec16a loss (Supplementary Fig. 6e, f). To increase the specificity of this finding in relation to neurodevelopment, the number of LysoTracker-positive punctae in a transgenic zebrafish line with Citrine-positive radial glial (RG) cells (Slc1a2b positive cells), marking RG and neuronal stem cells, was assessed. At 5 dpf, we observed a significant accumulation of lysosomes/autophagosomes in the radial processes of RG in *clec16a* crispants, indicating the need for Clec16a to maintain healthy neuronal progenitors during cortical development (Fig. 4c, d, ctrl = 4.8 *clec16a* = 9.0).

Microglia are brain macrophages, which are highly phagocytic in brain development, and require high lysosomal and membrane trafficking activity. In zebrafish, NDD-related genetic mechanisms have been linked to abnormalities in microglia and lysosomal activity in multiple brain cell types (Berdowski et al. 2022; Sanderson et al. 2021). To assess the contribution of microglial activation to CLEC16A-related embryonic development, we studied the number of microglia during developmental stages with the neutral red-uptake analysis. This test revealed that *clec16a* crispants have similar microglia numbers compared to control injected fish at 3 and 5 dpf (Supplementary Fig. 6 g–j). Despite the normal microglia numbers, *clec16*a crispants showed an increased number of acidic compartments in Mpeg1 + microglia at 5 dpf, as also seen in the RG cells (Fig. 4e, f).

To confirm that the lysosomal phenotype is caused by a lack of Clec16a, and that *CLEC16A*-Δ19 has perturbed activity in vivo, we performed rescue experiments using human *CLEC16A*-WT and *CLEC16A-*Δ19 mRNA. Co-injection of *CLEC16A*-WT mRNA at the single-cell stage in *clec16a* crispants showed a decrease in acidic compartments in Mpeg1 + cells compared to *clec16a* crispants (Fig. 4g, h). In contrast, injection of *CLEC16A*-Δ19 mRNA showed a slight increase in LysoTracker signal compared to *clec16a* crispants (H2O = 13.9, + WT = 11.0, + Δ19 = 16.3). These results suggest that *clec16a* defects cause accumulation of LysoTracker-positive acidic compartments in multiple brain cell types, and shows that, unlike *CLEC16A*-WT, *CLEC16A*-Δ19 fails to suppress this phenotype. This confirms that this variant perturbs functions of CLEC16A important in brain development, and supports that this is a pathogenic variant. Altogether, these data support a role for Clec16a during development in radial glia cells as well as immune-reactive microglia.

It has been shown that the CLEC16A/NRDP1/UPS8 complex regulates mitophagy through the NRDP1 target Parkin (Soleimanpour et al. 2014). Loss of murine Clec16a blocks turnover and proteasomal degradation of Parkin (Soleimanpour et al. 2014). We assessed the involvement of Clec16a-mediated mitophagy in embryonic brain development by evaluating the amount of unhealthy mitochondria in *clec16a* crispants. We stained mitochondria in zebrafish midbrain using MitoTracker™ Red CMXRos. We observed a pattern of organelles with increased MitoTracker intensity staining in the radial fibers of the RG in *clec16a* crispants at 5dpf, suggesting accumulation of unhealthy/damaged mitochondria (Fig. 4i) (Brunetti et al. 2012). We quantified the total area of the increased MitoTracker signal, which was a 2.4 fold higher (*p* = 0.0231) in the *clec16a* crispants compared to the control (Fig. 4j). In addition, immunoblots on whole fish lysates, showed a 2.3-fold increased Parkin abundance in the *clec16a* crispants (*p* = 0.0490), indicating failure of mitophagy control (Fig. 4k and Supplementary Fig. 7a, b). These results indicate that loss of Clec16a induces dysregulation of mitochondrial quality control, via inhibition of Parkin turnover, during embryonic brain development in zebrafish.

## Discussion

One of the several CLEC16A physiological functions is to protect against neurodegeneration. This report demonstrates the importance of CLEC16A also during brain development. We report deleterious variants in *CLEC16A* in association with a severe neurodevelopmental disorder. Our in vivo zebrafish model shows that Clec16a loss dysregulates autophagy and mitophagy already during brain development. Our in vitro studies provide new insights into CLEC16A’s physiological function by showing subcellular localization to early endosomes, interactions with TRIM27 and retromer complex protein VPS35, as well as alterations in (retromer dependent) processes that facilitate endosomal trafficking.

Several studies have shown consistent association of heterozygous *CLEC16A* SNPs with auto-immune disorders, particularly multiple sclerosis (International Multiple Sclerosis Genetics Consortium 2009; International Multiple Sclerosis Genetics Consortium et al. 2007; Nischwitz et al. 2011), type-1 diabetes (Hakonarson et al. 2007; Wellcome Trust Case Control Consortium 2007), and Crohn’s disease (Marquez et al. 2009), and that *CLEC16A* plays a role in the neurodegeneration of Parkinson disease (Fan et al. 2022; Strafella et al. 2021). We describe four probands from two unrelated families with bi-allelic *CLEC16A* loss-of-function variants who present with congenital and progressive microcephaly, brain atrophy, corpus callosum hypoplasia, and growth retardation. The two surviving children also showed a very similar postnatal course with hypotonia and severe developmental delay. Notably, they both suffered from severe infections and died with sepsis. The presence of three similarly affected pregnancies and segregation analysis in family 1 strongly suggests that the *CLEC16A* variant is causally related to the disease phenotype. The observation of a similarly affected patient from an unrelated family with a predicted pathogenic variant in *CLEC16A* makes LoF variants in this gene highly suspect for being the cause of an unrecognized recessive neurodevelopmental disorder.

Previous studies have shown that CLEC16A has a role in endosomal trafficking (van Luijn et al. 2015). Endosomes contain cargo that needs to be recycled to the plasma membrane, the trans-Golgi network or directed to late endosomes for degradation of their content in lysosomes (Elkin et al. 2016). Several protein complexes promote or prevent sorting in any of these directions. In previous research, CLEC16A has mainly been associated with recycling/late endosomes and interactions with the HOPS complex (van Luijn et al. 2015). Unexpectedly, we found localization to early endosomes under basal cell culture conditions in HEK293T cells. The physiological relevance of the early endosomal localization was supported by mass spectrometry data showing interactions with endosomal retromer complex components (VPS35/VPS26B) and E3-ubiquitin ligase TRIM27. Notably, several studies link VPS35 mutations and retromer to Parkinson disease (Ma et al. 2021; Rahman and Morrison 2019; Sassone et al. 2021). The TRIM27 protein is recruited to retromer-positive endosomes and forms a complex with MAGE-L2 and USP7 (Hao et al. 2013; Zhang et al. 2018). Together they regulate endosomal sorting via K63-linked ubiquitination of the WASH complex, which subsequently promotes the polymerization of new actin filaments at early endosomes (Hao et al. 2013, 2015).

Strict regulation of WASH ubiquitination and endosomal actin levels is essential for endosomal protein recycling (Hao et al. 2013, 2015). Since TRIM27 is one of the proteins involved in WASH ubiquitination and activation, there are several mechanisms that control TRIM27 protein levels. To promote its own degradation, TRIM27 can auto-ubiquitinate (Zaman et al. 2013). On the other hand, USP7 prevents TRIM27 auto-ubiquitination and subsequent degradation (Hao et al. 2013). While knockdown of USP7 and TRIM27 lowers F-actin production, TRIM27 mutants that are resistant to degradation accumulate F-actin, which is known to impair endosomal trafficking. We observed a similar accumulation of F-actin after downregulation of *CLEC16A* (Hao et al. 2015). Both impaired and enhanced F-actin polymerization impair proper endosomal trafficking, e.g., lead to defective mannose-6-phosphate receptor (M6PR) recycling (Hao et al. 2013). Although our results suggest that absence of CLEC16A induces increased TRIM27 and abnormal F-actin polymerization, follow-up studies are required to confirm abnormalities of the WASH/TRIM27-dependent endosomal trafficking. However, we hypothesize that the E3-ubiquitin ligase activity of CLEC16A contributes to the ubiquitination steps required for TRIM27 degradation and regulation of WASH complex activity.

One of the complexes that requires F-actin networks for its function is the retromer complex (Reitz 2018; Seaman 2021). Dysfunction of the retromer complex results in impaired endosomal trafficking of target proteins from endosomes to the Golgi, and endosome to cell surface (e.g., transferrin, B2 adrenoreceptor, GLUT1, ATP7A, a5B1, M6PR) (Seaman 2021). Moreover, retromer is involved in the delivery of cargo to the degradation pathway, and defects cause an accumulation of unhealthy mitochondria as well as an accumulation of autolysosomes (Reitz 2018; Seaman 2021). The consequences of defects in the degradative pathways, e.g., unhealthy mitochondria and (auto)lysosome accumulation, were observed in the developing zebrafish *clec16a* crispant brain and are in line with previously described features in adult CLEC16A KO/KD models(Kim and DiAntonio 2012; Pandey et al. 2018; Redmann et al. 2016; van Luijn et al. 2015).

In addition to the interaction with TRIM27, we observed the previously described interaction of CLEC16A with NRDP1, also known as RNF41. CLEC16A is described to stabilize the complex it forms with NRDP1 and USP8 by adding non-degradative ubiquitin conjugates to NRDP1 (Pearson et al. 2018). This ubiquitin-dependent complex regulates Parkin-mediated mitophagy in β-cells (Pearson et al. 2018). The observation of their interaction in HEK293T cells, together with the accumulation of unhealthy mitochondria in the developing zebrafish brains and increased Parkin levels, suggest that CLEC16A has a central role in the regulation of mitophagy during (brain) development. Most likely, CLEC16A mainly contributes to mitochondrial health during brain development by promoting PINK/PARKIN-dependent mitophagy via the well-established CLEC16A–NRDP1–USP8 complex (Pearson et al. 2018). However, we cannot rule out the presence of additional contributing pathways to the stimulation of retromer-dependent mitophagy processes, such as Bcl-xl translocation or transport of mitochondrial outer membrane proteins to mitochondria (Farmer et al. 2019).

Another mechanism that could contribute to the accumulation of mitochondria is dysregulation of autophagy. The contribution of CLEC16A to autophagic processes is well established in literature; however, Clec16a loss-of-function studies revealed defects in various autophagy stages including autophagy induction, endosomal maturation, and autolysosomal clearance (Kim et al. 2010, 2012; Pandey et al. 2021; Redmann et al. 2016; Team RC et al. 2017). We observed an accumulation of acidic phagolysosome compartments in radial glia cells and microglia during brain development, suggestive for involvement of the phagocytic process besides the mitophagy process.

Our observations point to the importance of autophagy and membrane trafficking during human brain development. The importance of WASH-mediated endosomal actin assembly and protein recycling during neurodevelopment has been suggested before. Variants in USP7 cause a neurodevelopmental disorder with developmental delay, behavioral, hypotonia, seizures, hypogonadism (MIM# 616,863) (Fountain et al. 2019; Hao et al. 2015). In addition, pathogenic MAGE-L2 variants result in the neurodevelopmental phenotypes of Chitayat–Hall, Schaaf–Yang (MIM# 615,547), and Prader–Willi (MIM# 176,270) syndromes (Buiting et al. 2014). Moreover, several independent studies indicate that the endosomal system is indispensable for normal brain development and function. Variants in multiple genes regulating endosomal trafficking and/or fusion of secretory vesicles [such as SMPD4, WDFY3, TRAPPC4, RAB18, VPS13B, EXOC7, EXOC8, VPS11 (Coulter et al. 2020; Le Duc et al. 2019; Magini et al. 2019; Momtazmanesh et al. 2020; Van Bergen et al. 2020)], have been associated with the occurrence of microcephaly, cerebral atrophy, and neurodevelopmental delay. Most of the corresponding proteins regulate autophagy and affect neuronal cell fate and survival.

Hyperactivity of the JAK/STAT pathway, with defective autophagic flux and increased ER stress, has been observed in *Clec16a* KO mice (Pandey et al. 2021). Abnormal neuroinflammation via sustained activation of the JAK/STAT pathway has also been implicated in the pathogenesis of the lethal cerebral pseudo-TORCH syndrome caused by deleterious *USP18* variants (Meuwissen et al. 2016). Both *Clec16a* KO mice and patients with *USP18* mutation respond with recovery and prolonged survival to immunomodulation by selective JAK/STAT inhibitors (Alsohime et al. 2020; Pandey et al. 2021). Future studies need to address the role of neuroinflammation in human brain of *CLEC16A* patients and the potential therapeutic value of JAK/STAT-selective anti-inflammatory drugs.

**Fig. 5:**
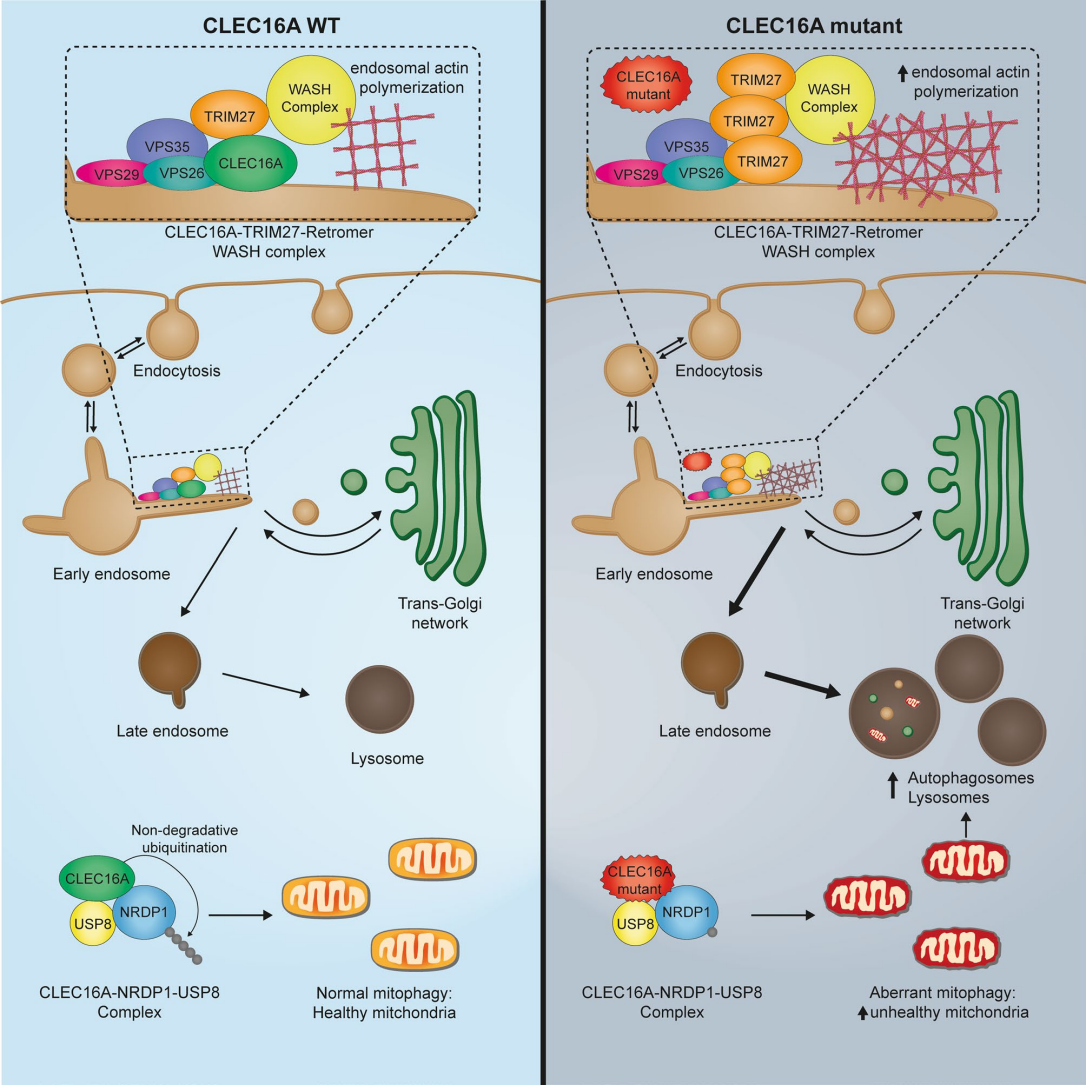
Graphical abstract of CLEC16A interaction with retromer and TRIM27. Left panel shows normal interaction of CLEC16A with early endosome, retromer, and TRIM27. Under physiological conditions, CLEC16A protects from autophagy and regulates mitophagy. Right panel: C-terminus truncated CLEC16A loses interaction with early endosomes, hereby increasing TRIM27 binding to early endosome and F-actin polymerization. CLEC16A dysfunction leads to dysregulated autophagy and accumulation of unhealthy mitochondria

In conclusion, our results highlight a role for E3-ubiquitin ligase CLEC16A in early endosomes by interacting with retromer components and by fine-tuning levels of TRIM27 and polymerized F-actin on the endosomal surface. These findings point to a role for CLEC16A in endosomal sorting, e.g., the maturation of early endosomes or retrograde transport to the trans-Golgi network or plasma membrane. Additionally, we show the relevance of CLEC16A-mediated mitophagy regulation during brain development via interactions with NRDP1 and regulation of PARKIN levels (Fig. 5). These observations build on the role of CLEC16A in protection against neurodegeneration, but at the same time provide new insights into the role of CLEC16A in human brain development. Given the diversity of functions described for CLEC16A, we hypothesize that CLEC16A has multiple ubiquitination targets, that can vary depending on the cell type, the developmental state, and the biological processes needed to regulate cellular homeostasis.

## Supplementary Information

Below is the link to the electronic supplementary material.

Supplementary file1 (PDF 1499 KB)

Supplementary file2 (XLSX 16 KB)

Supplementary file3 (XLSX 14 KB)

Supplementary file4 (XLSX 2165 KB)

Supplementary file5 (XLSX 2158 KB)

## Data Availability

All WES data are stored in a repository at the Clinical Genetics department of the Erasmus MC, the Netherlands or CENTOGENE, Germany.

## References

[CR1] Alsohime F, Martin-Fernandez M, Temsah MH, Alabdulhafid M, Le Voyer T, Alghamdi M, Qiu X, Alotaibi N, Alkahtani A, Buta S, Jouanguy E, Al-Eyadhy A, Gruber C, Hasan GM, Bashiri FA, Halwani R, Hassan HH, Al-Muhsen S, Alkhamis N, Alsum Z, Casanova JL, Bustamante J, Bogunovic D, Alangari AA (2020). JAK inhibitor therapy in a child with inherited USP18 deficiency. N Engl J Med.

[CR2] Barkovich AJ, Guerrini R, Kuzniecky RI, Jackson GD, Dobyns WB (2012). A developmental and genetic classification for malformations of cortical development: update 2012. Brain.

[CR3] Berdowski WM, van der Linde HC, Breur M, Oosterhof N, Beerepoot S, Sanderson L, Wijnands LI, de Jong P, Tsai-Meu-Chong E, de Valk W, de Witte M, van IJcken WFJ, Demmers J, van der Knaap MS, Bugiani M, Wolf NI, van Ham TJ (2022). Dominant-acting CSF1R variants cause microglial depletion and altered astrocytic phenotype in zebrafish and adult-onset leukodystrophy. Acta Neuropathol.

[CR4] Brinkman EK, Chen T, Amendola M, van Steensel B (2014). Easy quantitative assessment of genome editing by sequence trace decomposition. Nucleic Acids Res.

[CR5] Brunetti D, Dusi S, Morbin M, Uggetti A, Moda F, D'Amato I, Giordano C, d'Amati G, Cozzi A, Levi S, Hayflick S, Tiranti V (2012). Pantothenate kinase-associated neurodegeneration: altered mitochondria membrane potential and defective respiration in Pank2 knock-out mouse model. Hum Mol Genet.

[CR6] Buiting K, Di Donato N, Beygo J, Bens S, von der Hagen M, Hackmann K, Horsthemke B (2014). Clinical phenotypes of MAGEL2 mutations and deletions. Orphanet J Rare Dis.

[CR7] Coulter ME, Musaev D, DeGennaro EM, Zhang X, Henke K, James KN, Smith RS, Hill RS, Partlow JN, Muna A-S, Kamumbu AS, Hatem N, Barkovich AJ, Aziza J, Chassaing N, Zaki MS, Sultan T, Burglen L, Rajab A, Al-Gazali L, Mochida GH, Harris MP, Gleeson JG, Walsh CA (2020). Regulation of human cerebral cortical development by EXOC7 and EXOC8, components of the exocyst complex, and roles in neural progenitor cell proliferation and survival. Genet Med.

[CR8] D'Astolfo DS, Pagliero RJ, Pras A, Karthaus WR, Clevers H, Prasad V, Lebbink RJ, Rehmann H, Geijsen N (2015). Efficient intracellular delivery of native proteins. Cell.

[CR9] den Dunnen JT, Dalgleish R, Maglott DR, Hart RK, Greenblatt MS, McGowan-Jordan J, Roux AF, Smith T, Antonarakis SE, Taschner PE (2016). HGVS recommendations for the description of sequence variants: 2016 update. Hum Mutat.

[CR10] Elkin SR, Lakoduk AM, Schmid SL (2016). Endocytic pathways and endosomal trafficking: a primer. Wien Med Wochenschr.

[CR11] Ellett F, Pase L, Hayman JW, Andrianopoulos A, Lieschke GJ (2011). mpeg1 promoter transgenes direct macrophage-lineage expression in zebrafish. Blood.

[CR12] Fan HH, Cui L, Jiang XX, Song YD, Liu SS, Wu KY, Dong HJ, Mao M, Ovlyakulov B, Wu HM, Zhu JH, Zhang X (2022). Autoimmune disease associated CLEC16A variants convey risk of Parkinson's disease in Han Chinese. Front Genet.

[CR13] Farmer T, O'Neill KL, Naslavsky N, Luo X, Caplan S (2019). Retromer facilitates the localization of Bcl-xL to the mitochondrial outer membrane. Mol Biol Cell.

[CR14] Fountain MD, Oleson DS, Rech ME, Segebrecht L, Hunter JV, McCarthy JM, Lupo PJ, Holtgrewe M, Moran R, Rosenfeld JA, Isidor B, Le Caignec C, Saenz MS, Pedersen RC, Morgan TM, Pfotenhauer JP, Xia F, Bi W, Kang SL, Patel A, Krantz ID, Raible SE, Smith W, Cristian I, Torti E, Juusola J, Millan F, Wentzensen IM, Person RE, Kury S, Bezieau S, Uguen K, Ferec C, Munnich A, van Haelst M, Lichtenbelt KD, van Gassen K, Hagelstrom T, Chawla A, Perry DL, Taft RJ, Jones M, Masser-Frye D, Dyment D, Venkateswaran S, Li C, Escobar LF, Horn D, Spillmann RC, Pena L, Wierzba J, Strom TM, Parenti I, Kaiser FJ, Ehmke N, Schaaf CP (2019). Pathogenic variants in USP7 cause a neurodevelopmental disorder with speech delays, altered behavior, and neurologic anomalies. Genet Med.

[CR15] García-Cazorla A, Oyarzábal A, Saudubray J-M, Martinelli D, Dionisi-Vici C (2022). Genetic disorders of cellular trafficking trends in genetics. Trends Genet.

[CR16] Gingerich MA, Liu X, Chai B, Pearson GL, Vincent MP, Stromer T, Zhu J, Sidarala V, Renberg A, Sahu D, Klionsky DJ, Schnell S, Soleimanpour SA (2021). The human disease gene *CLEC16A* encodes an intrinsically disordered protein region required for mitochondrial quality control. bioRxiv.

[CR17] Hain HS, Pandey R, Bakay M, Strenkowski BP, Harrington D, Romer M, Motley WW, Li J, Lancaster E, Roth L, Grinspan JB, Scherer SS, Hakonarson H (2021). Inducible knockout of Clec16a in mice results in sensory neurodegeneration. Sci Rep.

[CR18] Hakonarson H, Grant SF, Bradfield JP, Marchand L, Kim CE, Glessner JT, Grabs R, Casalunovo T, Taback SP, Frackelton EC, Lawson ML, Robinson LJ, Skraban R, Lu Y, Chiavacci RM, Stanley CA, Kirsch SE, Rappaport EF, Orange JS, Monos DS, Devoto M, Qu HQ, Polychronakos C (2007). A genome-wide association study identifies KIAA0350 as a type 1 diabetes gene. Nature.

[CR19] Hao YH, Doyle JM, Ramanathan S, Gomez TS, Jia D, Xu M, Chen ZJ, Billadeau DD, Rosen MK, Potts PR (2013). Regulation of WASH-dependent actin polymerization and protein trafficking by ubiquitination. Cell.

[CR20] Hao YH, Fountain MD, Fon Tacer K, Xia F, Bi W, Kang SH, Patel A, Rosenfeld JA, Le Caignec C, Isidor B, Krantz ID, Noon SE, Pfotenhauer JP, Morgan TM, Moran R, Pedersen RC, Saenz MS, Schaaf CP, Potts PR (2015). USP7 acts as a molecular rheostat to promote WASH-dependent endosomal protein recycling and is mutated in a human neurodevelopmental disorder. Mol Cell.

[CR21] He C, Bartholomew CR, Zhou W, Klionsky DJ (2009). Assaying autophagic activity in transgenic GFP-Lc3 and GFP-Gabarap zebrafish embryos. Autophagy.

[CR22] Hetz C, Saxena S (2017). ER stress and the unfolded protein response in neurodegeneration. Nat Rev Neurol.

[CR23] Hirschfield GM, Xie G, Lu E, Sun Y, Juran BD, Chellappa V, Coltescu C, Mason AL, Milkiewicz P, Myers RP, Odin JA, Luketic VA, Bacon B, Bodenheimer H, Liakina V, Vincent C, Levy C, Pillai S, Lazaridis KN, Amos CI, Siminovitch KA (2012). Association of primary biliary cirrhosis with variants in the CLEC16A, SOCS1, SPIB and SIAE immunomodulatory genes. Genes Immun.

[CR24] International Multiple Sclerosis Genetics Consortium (2009). The expanding genetic overlap between multiple sclerosis and type I diabetes. Genes Immun.

[CR25] Hafler DA, Compston A, Sawcer S, Lander ES, Daly MJ, De Jager PL, de Bakker PI, Gabriel SB, Mirel DB, Ivinson AJ, Pericak-Vance MA, Gregory SG, Rioux JD, McCauley JL, Haines JL, Barcellos LF, Cree B, Oksenberg JR, Hauser SL, International Multiple Sclerosis Genetics Consortium (2007). Risk alleles for multiple sclerosis identified by a genomewide study. N Engl J Med.

[CR26] Jayaraman D, Bae BI, Walsh CA (2018). The genetics of primary microcephaly. Annu Rev Genom Hum Genet.

[CR27] Kim S, DiAntonio A (2012). A role for the membrane Golgi protein Ema in autophagy. Autophagy.

[CR28] Kim S, Wairkar YP, Daniels RW, DiAntonio A (2010). The novel endosomal membrane protein Ema interacts with the class C Vps-HOPS complex to promote endosomal maturation. J Cell Biol.

[CR29] Kim S, Naylor SA, DiAntonio A (2012). Drosophila Golgi membrane protein Ema promotes autophagosomal growth and function. Proc Natl Acad Sci U S A.

[CR30] Kizil C, Kaslin J, Kroehne V, Brand M (2012). Adult neurogenesis and brain regeneration in zebrafish. Dev Neurobiol.

[CR31] Kuil LE, Lopez Marti A, Carreras Mascaro A, van den Bosch JC, van den Berg P, van der Linde HC, Schoonderwoerd K, Ruijter GJG, van Ham TJ (2019). Hexb enzyme deficiency leads to lysosomal abnormalities in radial glia and microglia in zebrafish brain development. Glia.

[CR32] Kuil LE, Oosterhof N, Geurts SN, van der Linde HC, Meijering E, van Ham TJ (2019). Reverse genetic screen reveals that Il34 facilitates yolk sac macrophage distribution and seeding of the brain. Dis Model Mech.

[CR33] Le Duc D, Giulivi C, Hiatt SM, Napoli E, Panoutsopoulos A, Harlan De Crescenzo A, Kotzaeridou U, Syrbe S, Anagnostou E, Azage M, Bend R, Begtrup A, Brown NJ, Buttner B, Cho MT, Cooper GM, Doering JH, Dubourg C, Everman DB, Hildebrand MS, Santos FJR, Kellam B, Keller-Ramey J, Lemke JR, Liu S, Niyazov D, Payne K, Person R, Quelin C, Schnur RE, Smith BT, Strober J, Walker S, Wallis M, Walsh L, Yang S, Yuen RKC, Ziegler A, Sticht H, Pride MC, Orosco L, Martinez-Cerdeno V, Silverman JL, Crawley JN, Scherer SW, Zarbalis KS, Jamra R (2019). Pathogenic WDFY3 variants cause neurodevelopmental disorders and opposing effects on brain size. Brain.

[CR34] Li J, Jorgensen SF, Maggadottir SM, Bakay M, Warnatz K, Glessner J, Pandey R, Salzer U, Schmidt RE, Perez E, Resnick E, Goldacker S, Buchta M, Witte T, Padyukov L, Videm V, Folseraas T, Atschekzei F, Elder JT, Nair RP, Winkelmann J, Gieger C, Nothen MM, Buning C, Brand S, Sullivan KE, Orange JS, Fevang B, Schreiber S, Lieb W, Aukrust P, Chapel H, Cunningham-Rundles C, Franke A, Karlsen TH, Grimbacher B, Hakonarson H, Hammarstrom L, Ellinghaus E (2015). Association of CLEC16A with human common variable immunodeficiency disorder and role in murine B cells. Nat Commun.

[CR35] Ma KY, Fokkens MR, Reggiori F, Mari M, Verbeek DS (2021). Parkinson's disease-associated VPS35 mutant reduces mitochondrial membrane potential and impairs PINK1/Parkin-mediated mitophagy. Transl Neurodegener.

[CR36] Magini P, Smits DJ, Vandervore L, Schot R, Columbaro M, Kasteleijn E, van der Ent M, Palombo F, Lequin MH, Dremmen M, de Wit MCY, Severino M, Divizia MT, Striano P, Ordonez-Herrera N, Alhashem A, Al Fares A, Al Ghamdi M, Rolfs A, Bauer P, Demmers J, Verheijen FW, Wilke M, van Slegtenhorst M, van der Spek PJ, Seri M, Jansen AC, Stottmann RW, Hufnagel RB, Hopkin RJ, Aljeaid D, Wiszniewski W, Gawlinski P, Laure-Kamionowska M, Alkuraya FS, Akleh H, Stanley V, Musaev D, Gleeson JG, Zaki MS, Brunetti-Pierri N, Cappuccio G, Davidov B, Basel-Salmon L, Bazak L, Shahar NR, Bertoli-Avella A, Mirzaa GM, Dobyns WB, Pippucci T, Fornerod M, Mancini GMS (2019). Loss of SMPD4 causes a developmental disorder characterized by microcephaly and congenital arthrogryposis. Am J Hum Genet.

[CR37] Marquez A, Varade J, Robledo G, Martinez A, Mendoza JL, Taxonera C, Fernandez-Arquero M, Diaz-Rubio M, Gomez-Garcia M, Lopez-Nevot MA, de la Concha EG, Martin J, Urcelay E (2009). Specific association of a CLEC16A/KIAA0350 polymorphism with NOD2/CARD15(−) Crohn's disease patients. Eur J Hum Genet.

[CR38] Mero IL, Ban M, Lorentzen AR, Smestad C, Celius EG, Saether H, Saeedi H, Viken MK, Skinningsrud B, Undlien DE, Aarseth J, Myhr KM, Granum S, Spurkland A, Sawcer S, Compston A, Lie BA, Harbo HF (2011). Exploring the CLEC16A gene reveals a MS-associated variant with correlation to the relative expression of CLEC16A isoforms in thymus. Genes Immun.

[CR39] Meuwissen ME, Schot R, Buta S, Oudesluijs G, Tinschert S, Speer SD, Li Z, van Unen L, Heijsman D, Goldmann T, Lequin MH, Kros JM, Stam W, Hermann M, Willemsen R, Brouwer RW, Van IWF, Martin-Fernandez M, de Coo I, Dudink J, de Vries FA, Bertoli Avella A, Prinz M, Crow YJ, Verheijen FW, Pellegrini S, Bogunovic D, Mancini GM (2016). Human USP18 deficiency underlies type 1 interferonopathy leading to severe pseudo-TORCH syndrome. J Exp Med.

[CR40] Momtazmanesh S, Rayzan E, Shahkarami S, Rohlfs M, Klein C, Rezaei N (2020). A novel VPS13B mutation in Cohen syndrome: a case report and review of literature. BMC Med Genet.

[CR41] Murao N, Nishitoh H (2017). Role of the unfolded protein response in the development of central nervous system. J Biochem.

[CR42] Nischwitz S, Cepok S, Kroner A, Wolf C, Knop M, Muller-Sarnowski F, Pfister H, Rieckmann P, Hemmer B, Ising M, Uhr M, Bettecken T, Holsboer F, Muller-Myhsok B, Weber F (2011). More CLEC16A gene variants associated with multiple sclerosis. Acta Neurol Scand.

[CR43] Pandey R, Bakay M, Hain HS, Strenkowski B, Elsaqa BZB, Roizen JD, Kushner JA, Orange JS, Hakonarson H (2018). CLEC16A regulates splenocyte and NK cell function in part through MEK signaling. PLoS ONE.

[CR44] Pandey R, Bakay M, Strenkowski BP, Hain HS, Hakonarson H (2021). JAK/STAT inhibitor therapy partially rescues the lipodystrophic autoimmune phenotype in Clec16a KO mice. Sci Rep.

[CR45] Passemard S, Perez F, Gressens P, El Ghouzzi V (2019). Endoplasmic reticulum and Golgi stress in microcephaly. Cell Stress.

[CR46] Pearson G, Chai B, Vozheiko T, Liu X, Kandarpa M, Piper RC, Soleimanpour SA (2018). Clec16a, Nrdp1, and USP8 form a ubiquitin-dependent tripartite complex that regulates beta-cell mitophagy. Diabetes.

[CR47] Rahman AA, Morrison BE (2019). Contributions of VPS35 mutations to Parkinson's disease. Neuroscience.

[CR48] Redmann V, Lamb CA, Hwang S, Orchard RC, Kim S, Razi M, Milam A, Park S, Yokoyama CC, Kambal A, Kreamalmeyer D, Bosch MK, Xiao M, Green K, Kim J, Pruett-Miller SM, Ornitz DM, Allen PM, Beatty WL, Schmidt RE, DiAntonio A, Tooze SA, Virgin HW (2016). Clec16a is critical for autolysosome function and Purkinje cell survival. Sci Rep.

[CR49] Reitz C (2018). Retromer dysfunction and neurodegenerative disease. Curr Genom.

[CR50] Rijvers L, Melief MJ, van Langelaar J, van der Vuurst de Vries RM, Wierenga-Wolf AF, Koetzier SC, Priatel JJ, Jorritsma T, van Ham SM, Hintzen RQ, van Luijn MM (2020). The role of autoimmunity-related gene CLEC16A in the B cell receptor-mediated HLA class II pathway. J Immunol.

[CR51] Sanderson LE, Lanko K, Alsagob M, Almass R, Al-Ahmadi N, Najafi M, Al-Muhaizea MA, Alzaidan H, AlDhalaan H, Perenthaler E, van der Linde HC, Nikoncuk A, Kuhn NA, Antony D, Owaidah TM, Raskin S, Vieira L, Mombach R, Ahangari N, Silveira TRD, Ameziane N, Rolfs A, Alharbi A, Sabbagh RM, AlAhmadi K, Alawam B, Ghebeh H, AlHargan A, Albader AA, Binhumaid FS, Goljan E, Monies D, Mustafa OM, Aldosary M, AlBakheet A, Alyounes B, Almutairi F, Al-Odaib A, Aksoy DB, Basak AN, Palvadeau R, Trabzuni D, Rosenfeld JA, Karimiani EG, Meyer BF, Karakas B, Al-Mohanna F, Arold ST, Colak D, Maroofian R, Houlden H, Bertoli-Avella AM, Schmidts M, Barakat TS, van Ham TJ, Kaya N (2021). Bi-allelic variants in HOPS complex subunit VPS41 cause cerebellar ataxia and abnormal membrane trafficking. Brain.

[CR52] Sassone J, Reale C, Dati G, Regoni M, Pellecchia MT, Garavaglia B (2021). The role of VPS35 in the pathobiology of Parkinson's disease. Cell Mol Neurobiol.

[CR53] Schuster C, Gerold KD, Schober K, Probst L, Boerner K, Kim MJ, Ruckdeschel A, Serwold T, Kissler S (2015). The autoimmunity-associated gene CLEC16A modulates thymic epithelial cell autophagy and alters T cell selection. Immunity.

[CR54] Seaman MNJ (2021). The retromer complex: from genesis to revelations. Trends Biochem Sci.

[CR55] Siskos N, Stylianopoulou E, Skavdis G, Grigoriou ME (2021). Molecular genetics of microcephaly primary hereditary: an overview. Brain Sci.

[CR56] Skinningsrud B, Husebye ES, Pearce SH, McDonald DO, Brandal K, Wolff AB, Lovas K, Egeland T, Undlien DE (2008). Polymorphisms in CLEC16A and CIITA at 16p13 are associated with primary adrenal insufficiency. J Clin Endocrinol Metab.

[CR57] Skinningsrud B, Lie BA, Husebye ES, Kvien TK, Forre O, Flato B, Stormyr A, Joner G, Njolstad PR, Egeland T, Undlien DE (2010). A CLEC16A variant confers risk for juvenile idiopathic arthritis and anti-cyclic citrullinated peptide antibody negative rheumatoid arthritis. Ann Rheum Dis.

[CR58] Soleimanpour SA, Gupta A, Bakay M, Ferrari AM, Groff DN, Fadista J, Spruce LA, Kushner JA, Groop L, Seeholzer SH, Kaufman BA, Hakonarson H, Stoffers DA (2014). The diabetes susceptibility gene Clec16a regulates mitophagy. Cell.

[CR59] Soleimanpour SA, Ferrari AM, Raum JC, Groff DN, Yang J, Kaufman BA, Stoffers DA (2015). Diabetes susceptibility genes Pdx1 and Clec16a function in a pathway regulating mitophagy in beta-cells. Diabetes.

[CR60] Strafella C, Caputo V, Termine A, Assogna F, Pellicano C, Pontieri FE, Macchiusi L, Minozzi G, Gambardella S, Centonze D, Bossu P, Spalletta G, Caltagirone C, Giardina E, Cascella R (2021). Immune system and neuroinflammation in idiopathic Parkinson's disease: association analysis of genetic variants and miRNAs interactions. Front Genet.

[CR61] Szklarczyk D, Gable AL, Lyon D, Junge A, Wyder S, Huerta-Cepas J, Simonovic M, Doncheva NT, Morris JH, Bork P, Jensen LJ, Mering CV (2019). STRING v11: protein-protein association networks with increased coverage, supporting functional discovery in genome-wide experimental datasets. Nucleic Acids Res.

[CR62] Li MW, Gao YP, Pang YT, Yan S, Ge W, Lau CS, Chan VS, Team RC (2017). Human CLEC16A regulates autophagy through modulating mTOR activity. Exp Cell Res.

[CR63] Trujillano D, Bertoli-Avella AM, Kumar Kandaswamy K, Weiss ME, Koster J, Marais A, Paknia O, Schroder R, Garcia-Aznar JM, Werber M, Brandau O, Calvo Del Castillo M, Baldi C, Wessel K, Kishore S, Nahavandi N, Eyaid W, Al Rifai MT, Al-Rumayyan A, Al-Twaijri W, Alothaim A, Alhashem A, Al-Sannaa N, Al-Balwi M, Alfadhel M, Rolfs A, Abou Jamra R (2017). Clinical exome sequencing: results from 2819 samples reflecting 1000 families. Eur J Hum Genet.

[CR64] Van Bergen NJ, Guo Y, Al-Deri N, Lipatova Z, Stanga D, Zhao S, Murtazina R, Gyurkovska V, Pehlivan D, Mitani T, Gezdirici A, Antony J, Collins F, Willis MJH, Coban Akdemir ZH, Liu P, Punetha J, Hunter JV, Jhangiani SN, Fatih JM, Rosenfeld JA, Posey JE, Gibbs RA, Karaca E, Massey S, Ranasinghe TG, Sleiman P, Troedson C, Lupski JR, Sacher M, Segev N, Hakonarson H, Christodoulou J (2020). Deficiencies in vesicular transport mediated by TRAPPC4 are associated with severe syndromic intellectual disability. Brain.

[CR65] van Luijn MM, Kreft KL, Jongsma ML, Mes SW, Wierenga-Wolf AF, van Meurs M, Melief MJ, der Kant R, Janssen L, Janssen H, Tan R, Priatel JJ, Neefjes J, Laman JD, Hintzen RQ (2015). Multiple sclerosis-associated CLEC16A controls HLA class II expression via late endosome biogenesis. Brain.

[CR66] Vandervore LV, Schot R, Kasteleijn E, Oegema R, Stouffs K, Gheldof A, Grochowska MM, van der Sterre MLT, van Unen LMA, Wilke M, Elfferich P, van der Spek PJ, Heijsman D, Grandone A, Demmers JAA, Dekkers DHW, Slotman JA, Kremers GJ, Schaaf GJ, Masius RG, van Essen AJ, Rump P, van Haeringen A, Peeters E, Altunoglu U, Kalayci T, Poot RA, Dobyns WB, Bahi-Buisson N, Verheijen FW, Jansen AC, Mancini GMS (2019). Heterogeneous clinical phenotypes and cerebral malformations reflected by rotatin cellular dynamics. Brain.

[CR67] Vandervore LV, Schot R, Milanese C, Smits DJ, Kasteleijn E, Fry AE, Pilz DT, Brock S, Borklu-Yucel E, Post M, Bahi-Buisson N, Sanchez-Soler MJ, van Slegtenhorst M, Keren B, Afenjar A, Coury SA, Tan WH, Oegema R, de Vries LS, Fawcett KA, Nikkels PGJ, Bertoli-Avella A, Al Hashem A, Alwabel AA, Tlili-Graiess K, Efthymiou S, Zafar F, Rana N, Bibi F, Houlden H, Maroofian R, Person RE, Crunk A, Savatt JM, Turner L, Doosti M, Karimiani EG, Saadi NW, Akhondian J, Lequin MH, Kayserili H, van der Spek PJ, Jansen AC, Kros JM, Verdijk RM, Milosevic NJ, Fornerod M, Mastroberardino PG, Mancini GMS (2019). TMX2 Is a crucial regulator of cellular redox state, and its dysfunction causes severe brain developmental abnormalities. Am J Hum Genet.

[CR68] Wellcome Trust Case Control Consortium (2007). Genome-wide association study of 14,000 cases of seven common diseases and 3000 shared controls. Nature.

[CR69] Zaman MM, Nomura T, Takagi T, Okamura T, Jin W, Shinagawa T, Tanaka Y, Ishii S (2013). Ubiquitination-deubiquitination by the TRIM27-USP7 complex regulates tumor necrosis factor alpha-induced apoptosis. Mol Cell Biol.

[CR70] Zhang HX, Xu ZS, Lin H, Li M, Xia T, Cui K, Wang SY, Li Y, Shu HB, Wang YY (2018). TRIM27 mediates STAT3 activation at retromer-positive structures to promote colitis and colitis-associated carcinogenesis. Nat Commun.

