# Supplementary material for: CLEC16A interacts with retromer and TRIM27, and its loss impairs endosomal trafficking and neurodevelopment
Source: Hum Genet. 2022 Dec 20;142(3):379–97. doi: 10.1007/s00439-022-02511-3 (PMC9950183; doi:10.1007/s00439-022-02511-3)
Supplement: Supplementary file 1 — Supplementary file1 (PDF 1499 KB) [file 439_2022_2511_MOESM1_ESM.pdf]

CLEC16A interacts with retromer and TRIM27 and its loss impairs endosomal trafficking and neurodevelopment

Daphne J. Smits[1,6,*], Jordy Dekker[1,6,*], Rachel Schot[1], Brahim Tabarki[2], Amal Alhashem[2], Jeroen A.A. Demmers[3], Dick H.W. Dekkers[3], Antonio Romito[4], Peter J van der Spek[5], Tjakko J. van Ham[1], Aida M. Bertoli-Avella[4], Grazia M.S. Mancini[1]

Human genetics

Corresponding author: Daphne J. Smits; e-mail address: d.smits@erasmusmc.nl, Phone number: +31107033764

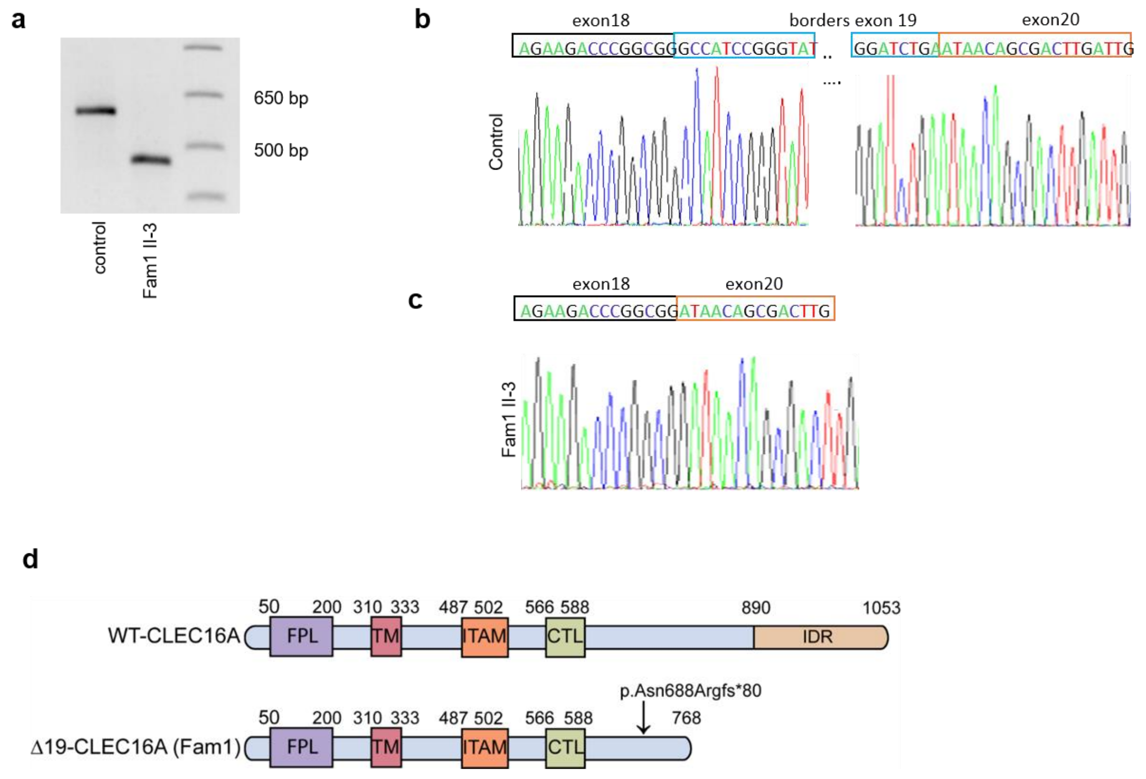

Supplementary Fig. 1 Genomic analysis of the *CLEC16A* variant found in family 1.
a Result of RT-PCR on cDNA showing a shorter *CLEC16A* transcript in subject II-3 fam1. The predicted product size is 586 base pairs in the control sample. Deletion of exon 19 will result in a product of 465 base pairs. **b, c** Sanger sequencing results showing the control sequence (**b**) and the homozygous deletion of exon 19 on cDNA derived from skin fibroblasts of individual II-3 fam 1 (**c**). **d** Schematic overview of the WT-CLEC16A and CLEC16A-Δ19 protein, showing the known functional domains (FPL, TM: transmembrane domain, ITAM: immunoreceptor tyrosine-based activation motif, CTL: C-type lectin domain).

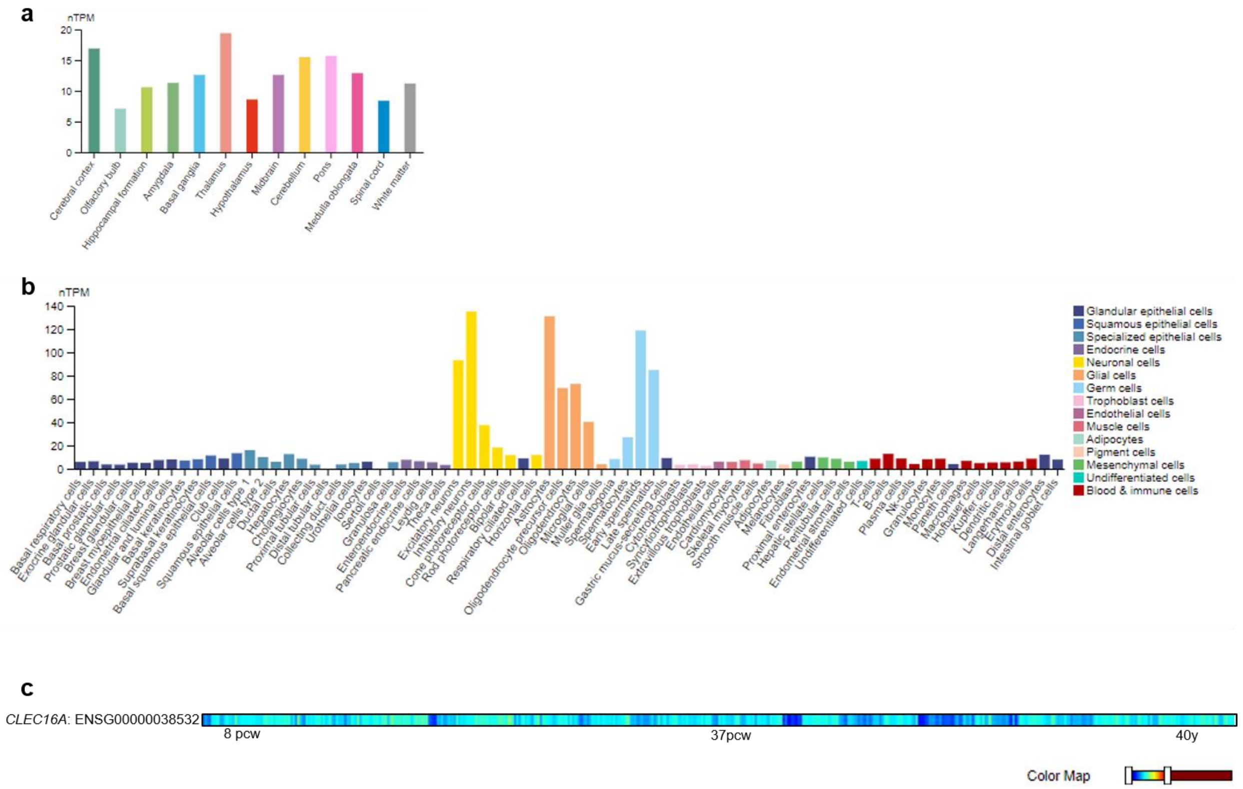

Supplementary Fig. 2 *CLEC16A* RNA and protein expression data **a** Plot from the Human Protein Atlas (https://www.proteinatlas.org/) showing *CLEC16A* expression throughout different tissues in the central nervous system. (nTPM: normalized transcripts per million) **b** Human protein atlas plot showing expression of *CLEC16A* in various tissues. **c** *CLEC16A* expression in the brain during different stages of development. Plot was adapted from the Allen brain atlas (https://portal.brain-map.org/) (pcw: post conceptional week; y: year).

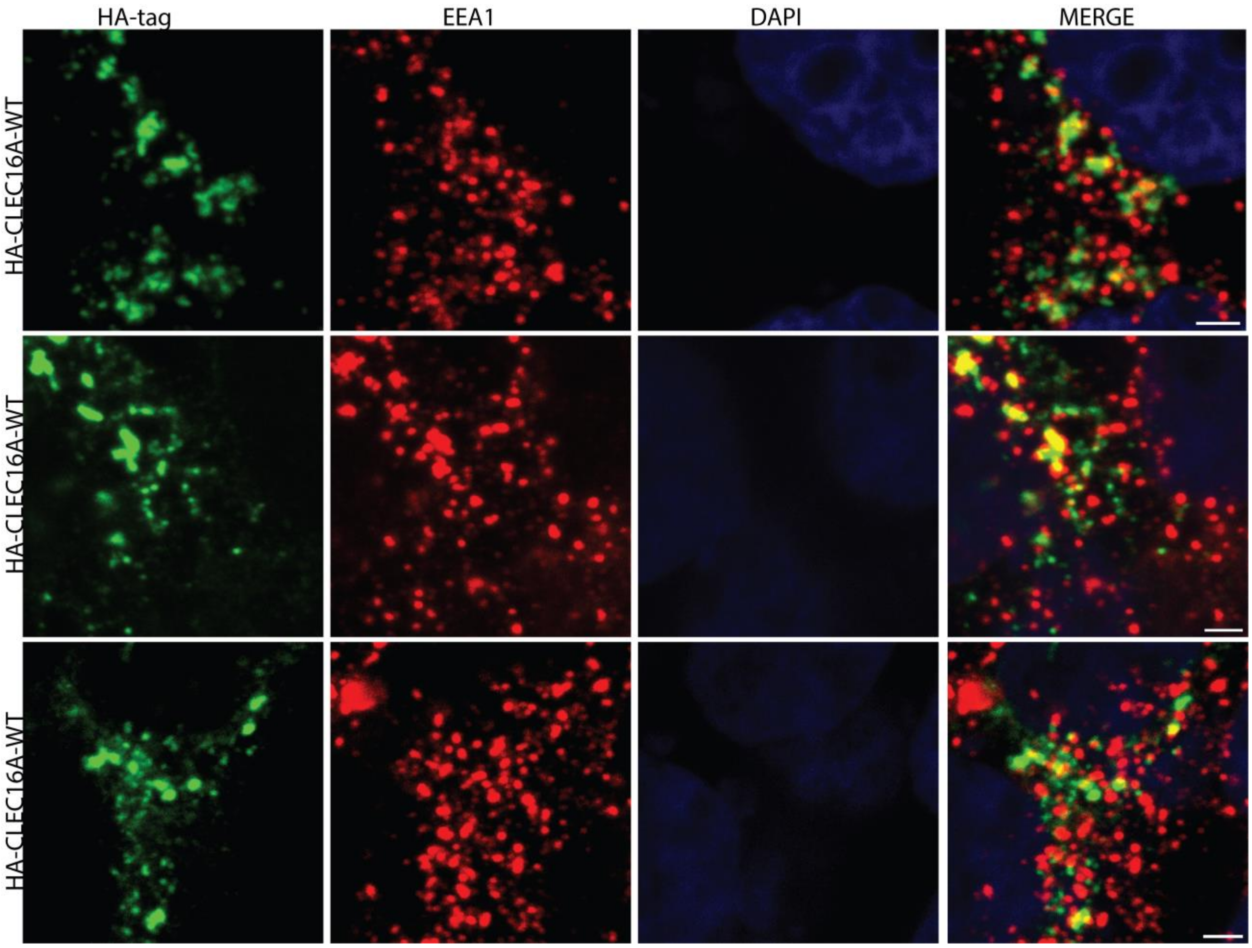

Supplementary Fig. 3 Co-localization of HA-CLEC16A with early endosomes. Immunocytochemistry assessing the localization of transiently expressed N-terminally tagged HA-CLEC16A-WT (green) in HEK293T cells 48 hours after transfection. Cells were stained with the early endosomal marker EEA1 (red), and antibodies directed against the HA-tag (green). Nuclei were counterstained with DAPI (blue). The images show in each row three representative cells from two replicate experiments. Images were made with confocal microscopy. Scale bars represent 2 μm.

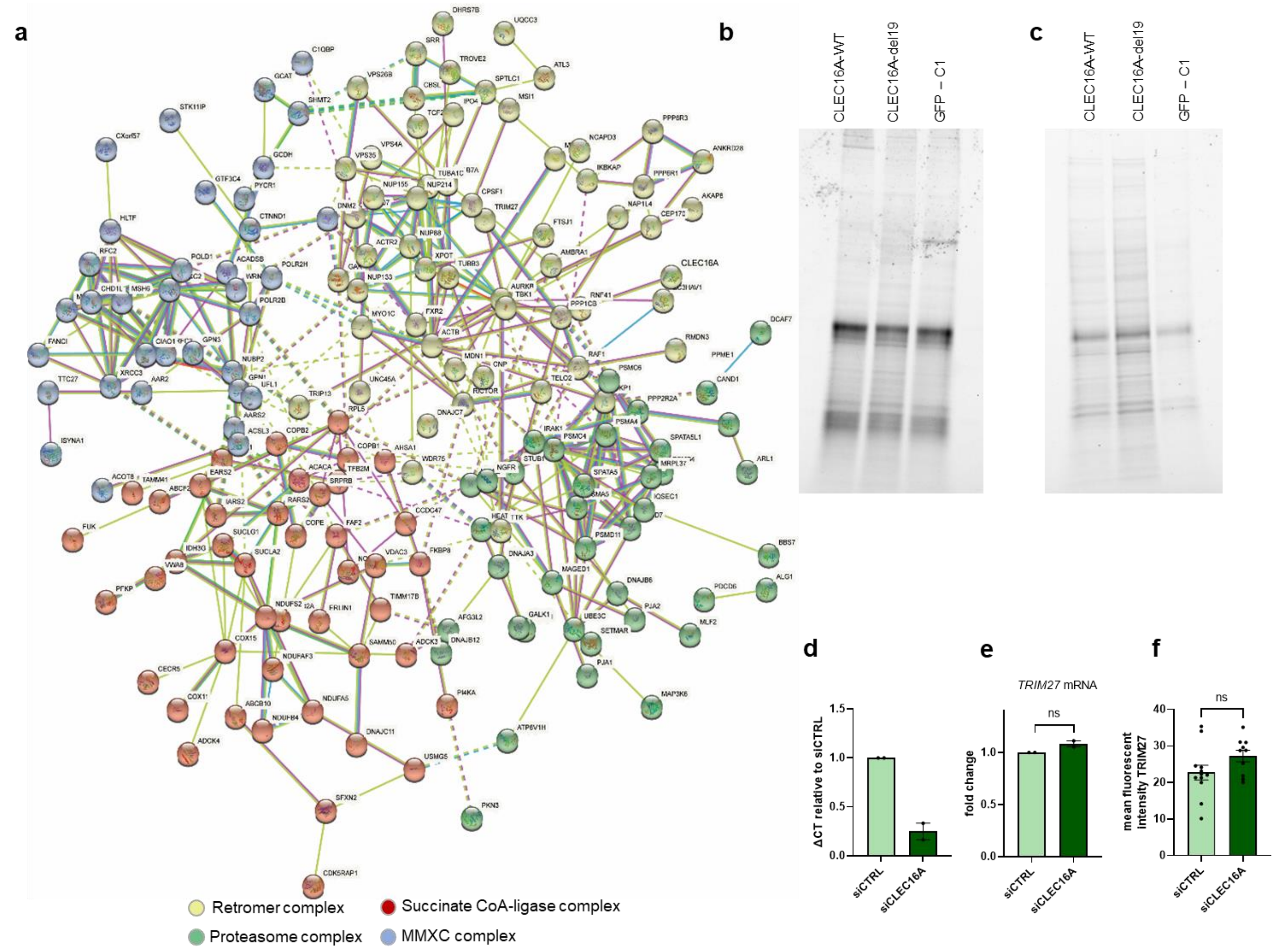

Supplementary Fig. 4 Subcellular compartment analysis of the CLEC16A interactome. **a** Subcellular compartment analysis on CLEC16A interactors identified with Mass spectrometry after pulldown of WT-CLEC16A-GFP from HEK293T cells. All interactors with an Andromeda score >40 were uploaded into the STRING platform. Functional protein clustering was performed within the platform with the K-means clustering option, displaying four clusters with a PPI enrichment P-value <1.0^{E-16}. Functional enrichment for cellular compartments was assessed on the four identified clusters, FDR<0.05. **b, c** Stain-free gels used as loading control for the western blots showing interaction of CLEC16A with VPS35 (B, belonging to Fig3B) and TRIM27 (C, belonging to Fig3C) in HEK293T cells following on immunoprecipitations with anti-GFP. **d** The efficiency of the siRNA smart pool against *CLEC16A* was tested in HEK293T cells with qRT-PCR 24h after transfection (n=2 experiments). **e** The effect of CLEC16A depletion on *TRIM27* RNA was determined with qRT-PCR (n=2 experiments, fold change 1.08, unpaired t-test with Welch's correction). **f** The effect of CLEC16A depletion of TRIM27 protein was assessed by calculating the mean fluorescent intensity (MFI) after immunostaining with TRIM27. MFI in siCTRL=22.7, MRI in siCLEC16A=27.5 (n=2 experiments; n=12-15 fields/section, unpaired t-test with Welch's correction).

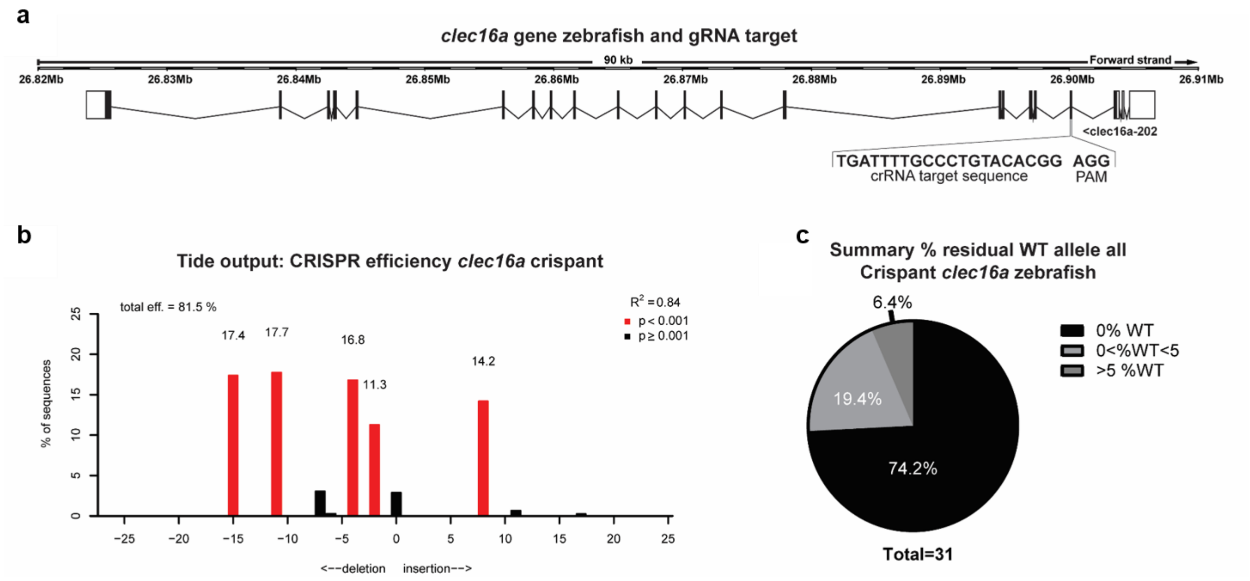

Supplementary Fig. 5 *clec16a* Crispants generation and efficiency. **a** Schematic representation of *clec16a* gene (Ensembl transcript ID: ENSDARG00000038094). The crRNA sequences is targeted to exon 4 of *clec16a*. **b** Indel spectrum of one CRISPR-Cas9 injected zebrafish embryo showing various types of indels with no detection of a WT allele. Plot was generated using TIDE. **c** Summary of the percentages of WT allele found in 31 *clec16a* Crispant zebrafish. Results shown are from four independent CRISPR-Cas9 injection rounds.

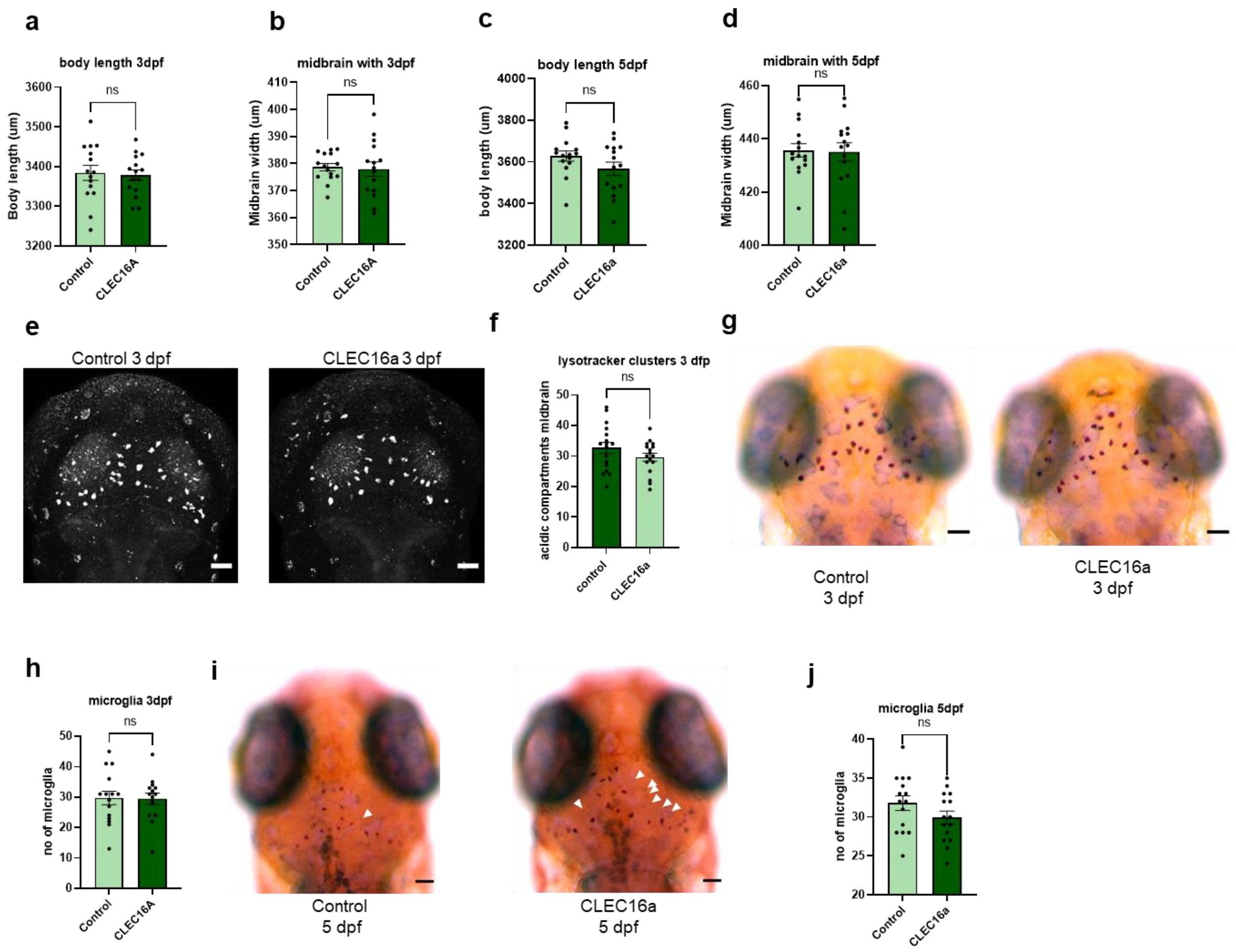

Supplementary Fig. 6 Microglia quantification and growth measurements in *clec16a* crispants.
a Body length (µm) of control and *CLEC16a* at 3 dpf. **b** Midbrain width (µm) at 3 dpf; the midbrain width was determined by measuring the largest diameter of the midbrain. **c,d** Same as A-B but then at 5 dpf. **e, f** Number of acidic compartments with a size > 5 µm, in the optic tectae of *clec16a* crispant zebrafish brain at 3 dpf. Scale bar represents 50µm (n=2 experiments, unpaired t-test with Welch's correction). **g, j** Images of 3 dpf/5dpf control and *clec16a* crispant zebrafish brain after neutral red staining. The red spots indicate the neutral red positive microglia in the midbrain. For both 3 and 5 dpf: n=15 fish, N=1 experiment. Unpaired t-test with Welch's correction. White arrows in indicate the smaller neutral red inclusions that are not microglia but other cell types. Scale bars in this figure represent 50 µm.

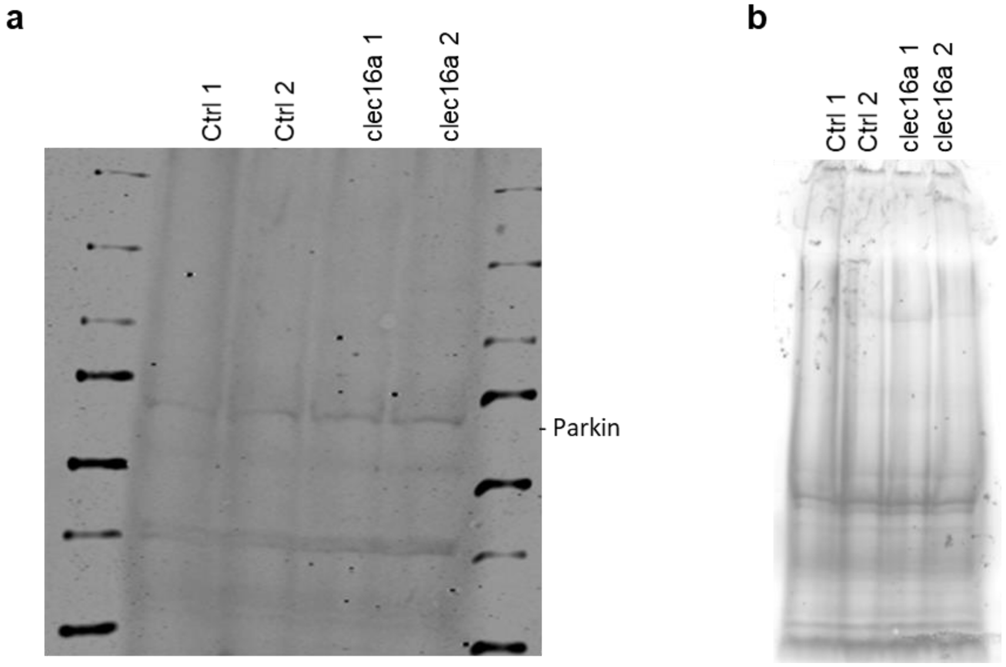

Supplementary Fig. 7 Parkin immunoblot on *clec16a* crispants. **a** Immunoblot of control injected zebrafish or *clec16a* crispants at 5 dpf in WT AB background. Parkin abundance is increased in *clec16a* crispants for both biological replicates (each lane represents a lysate of 15 fish, the numbers 1 and 2 represent two different zebrafish breedings; Parkin: 55kDa). **b** Stain-free gels used as loading control for the western blots on whole control injected zebrafish and *clec16a* crispants (belonging to Fig. 4k). (each lane represents a lysate of 15 fish, the numbers 1 and 2 represent two different zebrafish breedings)

Supplementary Table 1 Primer sequences used in this manuscript.

**Primer sequences:**
**RT-PCR**
CLEC16A_RT_FW:GAGAAGAAAGTGTTCACCTTGTACGAC
CLEC16A_RT_RV:ATGGGGAAGGGCTTGGAATG
**Site directed mutagenesis**
Q5SDM _del_ex19_CLEC16_F: ATAACAGCGACTTGATTGCATGTACAG
Q5SDM _del_ex19_CLEC16_ R: CCGCCGGGTCTTCTCCAC
**Sanger sequencing plasmids**
CLEC16A_GFP_WT_FW1: TCCTGCTGGAGTTCGTGACC
CLEC16A_GFP_WT_RV1: TGATGGAACGGATGGTCTCC
CLEC16A_GFP_WT_FW2: CGCAACATCCACTCCTTGG
CLEC16A_GFP_WT_RV2: CGTTCCTGAAAACACTTTCGTT
CLEC16A_GFP_WT_FW3: TTCATAAATTTGACTTTTCTGATGAGG
CLEC16A_GFP_WT_RV3: CACATGGCTCCCAATGAACC
CLEC16A_GFP_WT_FW4: GGCCATGCTGCACTACATCC
CLEC16A_GFP_WT_RV4: GCAGGAAGAGCCTGTTGAGC
CLEC16A_GFP_WT_FW5: GCTCTTCCTGCCCCTCTACG
CLEC16A_GFP_WT_RV5: CCTTCTCGGCGTCTTCTTGG
CLEC16A_GFP_WT_FW6: GTGGCGATGTGGAGAAGACC
CLEC16A_GFP_WT_RV6: GGTGACTGGCGTGGAGGAC
CLEC16A_GFP_WT_FW7: GCGGGTGCAAAAGAGACC
CLEC16A_GFP_WT_RV7: TGGCATAGAGGAGGCAGAGC
CLEC16A_GFP_WT_FW8: CCACGGACGAGGAGAAAAGC
CLEC16A_GFP_WT_RV8: GCAGCCAGCACTCATCAGG
CLEC16A_GFP_WT_FW9: CCAGCCAGATGGGAAGATCC
CLEC16A_GFP_WT_RV9: CTCGCAATTGCAGTGACAGG
CLEC16A_GFP_WT_FW10: TGGGGAGTGGTCAAGTTTGC
CLEC16A_GFP_WT_RV10: GAAAGGCAGGTGCTGAGTGG
CLEC16A_GFP_WT_FW11: CACTCAGCACCTGCCTTTCC
CLEC16A_GFP_WT_RV11: GTTCAGGGGGAGGTGTGG
**Zebrafish related experiments**
**mRNA synthesis for rescue experiments**
CLEC16A_T7_FW: AATTAATACGACTCACTATAGGGCCACCATGTTTGGCCGCTCGC
CLEC16A_T7_RV: GCTTTATTTGTAACCATTATAAGCTGC
**Genotyping zebrafish larvae**
Clec16a_fw_DR: 5’-TTGCATTCAGGGACTATTTAGTGG-3’
Clec16a_rv_DR: 5’-GTTACTCACGGATGACAGAGAGCAG-3’

Supplementary Table 4: Prediction score of alternative initiation codons in family 2 by ATGpr.

No of ATG from 5’end	reliability	Identity to Kozak rule (A/GxxATGG)	Start (bp)	Finish (bp)	ORF length (AA)	Stop codon found?	Sequence
**2**	0.67	GXXATGt	138	2855	906	Yes	MFGRSRSWVGGGHGKTSRNIHSLDHLKYLYHVLTKNTTVTEQNRNLLVETIRSITEIL IWGDQNDSSVFDFFLEKNMFVFFLNILRQKSGRYVCVQLLQTLNILFENISHETSLYYL LSNNYVNSIIVHKFDFSDEEIMAYYISFLKTLS LKLNNHTVHFFYNEHTNDFALYTEAIKFFNHPESMVRIAVRTITLNVYKV DNQAMLHYIRDKTAVPYFSNLVWFIGSHVIELDDCVQTDEEHRNRGKLSDLVAEHL DHLHYLNDILIINCEFLNDVLTDHLLNRLFLPLYVYSLENQDKG GERPKISLPVSLYLLSQVFLIIHHAPLVNSLAEVILNGDLSEMYAKTEQD IQRSSAKPSIRCFIKPTETLERSLEMNKHKGKRRVQKRPNYKNVGEEEDE EKGPTEDAQEDAEKAKEIEMVIMERSKLSELAASTSVQEQNTTDEEKSAA ATCSESTQWSRPFLDMVYHALDSPDDDYHALFVLCLLYAMSHNKGMDPEKLERIQL PVPNAAEKTTYNHPLAERLIRIMNNAAQPDGKIRLATLELSCLL LKQQVLMSAGCIMKDVHLACLEGAREESVHLVRHFYKGEDIFLDMFEDEYRSMTM KPMNVEYLMMDASILLPPTGTPLTGIDFVKRLPCGDVEKTRRAIRVFFMLRSLSLQLR GEPETQLPLTREEDLIKTDDVLDLNNSDLIACTVITK DGGMVQRFLAVDIYQMSLVEPDVSRLGWGVVKFAGLLQDMQVTGVEDDSRALNI TIHKPASSPHSKPFPILQATFIFSDHIRCIIAKQRLAKGRIQARRMKMQRIAALLDLPIQ PTTEVLGFGLGSSTSTQHLPFRFYDQGRRGSSDPTVQRSVFASVDKVPGEPAPRPAP QLVHHGGRSRSFSLWSLCELPFLSQKPRRLAAPAS
**10**	0.41	AXXATGG	690	2855	722	Yes	MVRIAVRTITLNVYKVDNQAMLHYIRDKTAVPYFSNLVWFIGSHVIELDD CVQTDEEHRNRGKLSDLVAEHLDHLHYLNDILIINCEFLNDVLTDHLLNR LFLPLYVYSLENQDKGGERPKISLPVSLYLLSQVFLIIHHAPLVNSLAEV ILNGDLSEMYAKTEQDIQRSSAKPSIRCFIKPTETLERSLEMNKHKGKRR VQKRPNYKNVGEEEDEEKGPTEDAQEDAEKAKEIEMVIMERSKLSELAAS TSVQEQNTTDEEKSAAATCSESTQWSRPFLDMVYHALDSPDDDYHALFVLCLLYAM SHNKGMDPEKLERIQLPVPNAAEKTTYNHPLAERLIRIMNNAAQPDGKIRLATLELS CLLLKQQVLMSAGCIMKDVHLACLEGAREESVHLVRH FYKGEDIFLDMFEDEYRSMTMKPMNVEYLMMDASILLPPTGTPLTGIDFV KRLPCGDVEKTRRAIRVFFMLRSLSLQLRGEPETQLPLTREEDLIKTDDV LDLNNSDLIACTVITKDGGMVQRFLAVDIYQMSLVEPDVSRLGWGVVKFAGLLQD MQVTGVEDDSRALNITIHKPASSPHSKPFPILQATFIFSDHIRCI IAKQRLAKGRIQARRMKMQRIAALLDLPIQPTTEVLGFGLGSSTSTQHLP FRFYDQGRRGSSDPTVQRSVFASVDKVPGEPAPRPAPQLVHHGGRSRSFS LWSLCELPFLSQKPRRLAAPAS
**21**	0.34	GXXATGa	1263	2855	531	Yes	MNKHKGKRRVQKRPNYKNVGEEEDEEKGPTEDAQEDAEKAKEIEMVIMERSKLSE LAASTSVQEQNTTDEEKSAAATCSESTQWSRPFLDMVYHALDSPDDDYHALFVLCL LYAMSHNKGMDPEKLERIQLPVPNAAEKTTYNHPLAERLIRIMNNAAQPDGKIRLA TLELSCLLLKQQVLMSAGCIMKDVHLACLEGAREESVHLVRHFYKGEDIFLDMFEDE YRSMTMKPMNVEYLMMDASILLPPTGTPLTGIDFVKRLPCGDVEKTRRAIRVFFML RSLSLQLRGEPETQLPLTRE EDLIKTDDVLDLNNSDLIACTVITKDGGMVQRFLAVDIYQMSLVEPDVSR LGWGVVKFAGLLQDMQVTGVEDDSRALNITIHKPASSPHSKPFPILQATF IFSDHIRCIIAKQRLAKGRIQARRMKMQRIAALLDLPIQPTTEVLGFGLG SSTSTQHLPFRFYDQGRRGSSDPTVQRSVFASVDKVPGEPAPRPAPQLVH HGGRSRSFSLWSLCELPFLSQKPRRLAAPAS
**35**	0.29	AXXATGa	1722	2855	378	Yes	MNNAAQPDGKIRLATLELSCLLLKQQVLMSAGCIMKDVHLACLEGAREES VHLVRHFYKGEDIFLDMFEDEYRSMTMKPMNVEYLMMDASILLPPTGTPLTGIDFV KRLPCGDVEKTRRAIRVFFMLRSLSLQLRGEPETQLPLTREEDL IKTDDVLDLNNSDLIACTVITKDGGMVQRFLAVDIYQMSLVEPDVSRLGW GVVKFAGLLQDMQVTGVEDDSRALNITIHKPASSPHSKPFPILQATFIFS DHIRCIIAKQRLAKGRIQARRMKMQRIAALLDLPIQPTTEVLGFGLGSST STQHLPFRFYDQGRRGSSDPTVQRSVFASVDKVPGEPAPRPAPQLVHHGGRSRSFS LWSLCELPFLSQKPRRLAAPAS
**39**	0.28	GXXATGt	1920	2855	312	Yes	MFEDEYRSMTMKPMNVEYLMMDASILLPPTGTPLTGIDFVKRLPCGDVEKTRRAIR VFFMLRSLSLQLRGEPETQLPLTREEDLIKTDDVLDLNNSDLIA CTVITKDGGMVQRFLAVDIYQMSLVEPDVSRLGWGVVKFAGLLQDMQVTGVEDD SRALNITIHKPASSPHSKPFPILQATFIFSDHIRCIIAKQRLAKGR IQARRMKMQRIAALLDLPIQPTTEVLGFGLGSSTSTQHLPFRFYDQGRRG SSDPTVQRSVFASVDKVPGEPAPRPAPQLVHHGGRSRSFSLWSLCELPFL SQKPRRLAAPAS

Supplementary Table 6: DAVID cellular compartment analysis: WT-CLEC16A-GFP

GO term CC	P-value	Benjamini
Mitochondrion	3.3^{E-14}	1.0^{E-11}
Membrane	1.2^{E-12}	1.9^{E-10}
Cytosol	1.7^{E-10}	1.7^{E-8}
Mitochondrial inner membrane	3.6^{E-9}	2.8^{E-7}
Cytoplasm	1.8^{E-7}	1.1^{E-5}
Mitochondrial matrix	1.4^{E-6}	7.3^{E-5}
Nucleoplasm	2.1^{E-6}	9.4^{E-5}
Nuclear pore	4.2^{E-6}	1.6^{E-4}
Proteasome complex	1.2^{E-5}	4.2^{E-4}
Retromer complex	1.7^{E-4}	5.2^{E-3}
Endoplasmic reticulum membrane	3.8^{E-4}	1.1^{E-2}
COPI vesicle coat	7.1^{E-4}	1.8^{E-2}
Mitochondrial outer membrane	7.5^{E-4}	1.8^{E-2}
Extracellular exosome	1.7^{E-3}	3.8^{E-2}
Nuclear membrane	3.2^{E-2}	6.5^{E-2}
Condensed chromosome, centromeric region	3.4^{E-3}	6.5^{E-2}

Supplementary Table 7: DAVID cellular compartment analysis: Interactors that lost binding to Δ19-CLEC16A-GFP

GO term CC	P-value	Benjamini
Nuclear pore	6.5^{E-6}	6.0^{E-4}
Nuclear membrane	3.9^{E-6}	1.8^{E-3}
Nuclear envelope	2.4^{E-3}	6.0^{E-2}
Membrane	2.6^{E-3}	6.0^{E-2}
